# Supplementary material for: Impact of ammonia levels on outcome in clinically stable outpatients with advanced chronic liver disease
Source: JHEP Rep. 2023 Jan 23;5(4):100682. doi: 10.1016/j.jhepr.2023.100682 (PMC9976454; doi:10.1016/j.jhepr.2023.100682)
Supplement: Multimedia component 1 [file mmc1.pdf]

Impact of ammonia levels on outcome in clinically stable outpatients with advanced chronic liver disease

Lorenz Balcar, Julia Krawanja, Bernhard Scheiner, Rafael Paternostro, Benedikt Simbrunner, Georg Semmler, Mathias Jachs, Lukas Hartl, Albert Friedrich Stättermayer, Philipp Schwabl, Matthias Pinter, Thomas Szekeres, Michael Trauner, Thomas Reiberger, Mattias Mandorfer

Table of contents

Supplementary results 2

Fig. S1 3

Fig. S2 5

Table S1 6

Table S2 8

Table S3 10

Table S4 ....**Error! Bookmark not defined.**

Table S5 ....**Error! Bookmark not defined.**

Table S6 ....**Error! Bookmark not defined.**

Table S7 ....**Error! Bookmark not defined.**

Table S8 20

Supplementary results

Associations between ammonia and biomarkers in compensated/decompensated ACLD patients in the pathophysiology cohort

As demonstrated in Figure 5, ammonia showed low correlations with UNOS MELD (2016)-score (Spearman's $\rho=0.361$; $p=0.002$), HVPG ($\rho=0.417$; $p<0.001$), VWF ($\rho=0.452$; $p<0.001$), ELF-test ($\rho=0.426$; $p<0.001$), IL-6 ($\rho=0.308$; $p=0.008$), and BA ($\rho=0.406$; $p<0.001$), as well as associations with CRP ($\rho=0.270$; $p=0.020$), and LBP ($\rho=-0.268$; $p=0.021$) in patients with compensated ACLD.

In patients with decompensated ACLD, ammonia showed low correlation with UNOS MELD (2016)-score (Spearman's $\rho=0.372$; $p<0.001$), HVPG ($\rho=0.385$; $p<0.001$), VWF ($\rho=0.314$; $p<0.001$), and BA ($\rho=0.395$; $p<0.001$), as well as associations with PCT ($\rho=0.214$; $p=0.020$), ELF-test ($\rho=0.280$; $p=0.002$) and serum sodium ($\rho=-0.197$; $p=0.031$).

Supplementary figures

Fig. S1

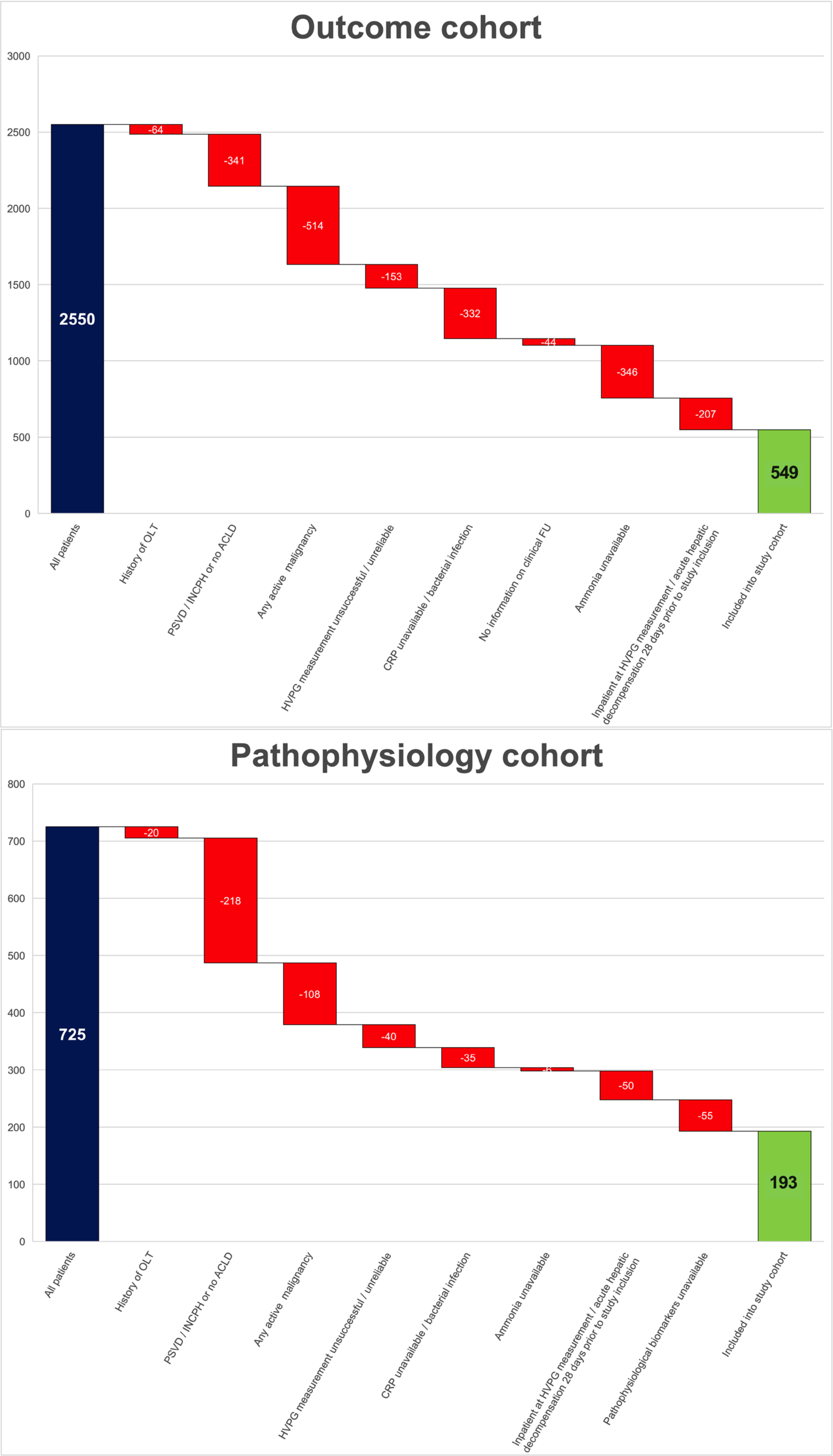

Fig. S1. Patient flow-chart showing the application of in- and exclusion.

Abbreviations: ACLD advanced chronic liver disease; CRP C-reactive protein; FU follow-up; HVPG hepatic venous pressure gradient; INCPH idiopathic non-cirrhotic portal hypertension; PSVD porto-sinusoidal vascular disorder; OLT orthotopic liver transplantation

Fig. S2

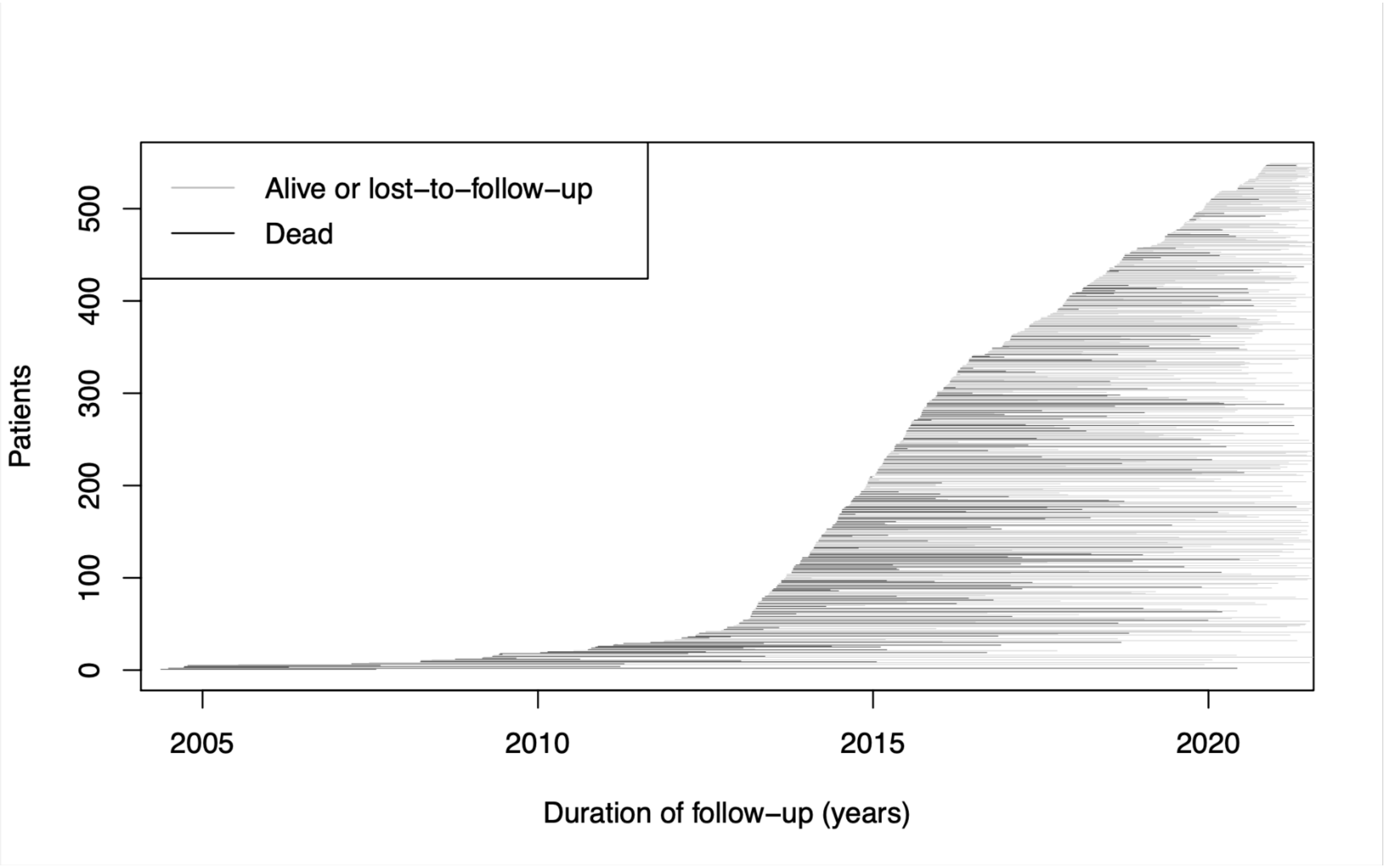

Fig. S2. Recruitment and follow-up over the study period.

Supplementary tables

Table S1

Disease severity indices	**Number of patients, n (%)**	**NH3, μmol x L^{-1}, median (IQR)**	**p-value**
CTP score			
A	342 (62%)	33.8 (25.8-45.3)	**<0.001**
B	168 (31%)	44.9 (35.8-58.8)	
C	39 (7%)	52.3 (37.4-80.3)	
UNOS MELD (2016) score			
<10	208 (38%)	31.3 (23.4-40.0)	**<0.001**
10-14	216 (39%)	41.5 (30.5-54.8)	
≥15	125 (23%)	46.4 (36.5-64.5)	
HVPG, mmHg			
HVPG 0-5 mmHg	42 (8%)	28.3 (24.0-37.1)	**<0.001**
HVPG 6-9 mmHg	83 (15%)	30.7 (22.0-35.8)	
HVPG 10-15 mmHg	137 (25%)	35.5 (26.8-46.0)	
HVPG ≥16 mmHg	287 (52%)	44.8 (33.8-59.6)	
Clinical Stages			
Probable cACLD	42 (8%)	28.3 (24.0-37.1)	**<0.001**
CS 0	74 (14%)	29.8 (21.7-35.2)	
CS 1	185 (34%)	35.8 (27.6-47.1)	
CS 2	25 (5%)	45.3 (33.7-60.0)	
CS 3	132 (24%)	40.1 (30.1-56.9)	
CS 4	91 (17%)	49.9 (40.3-68.7)	
Substages of ACLD			
Compensated	297 (54%)	33.6 (25.6-43.1)	-
SDC	209 (38%)	45.8 (34.7-62.0)	0.934
UDC	36 (7%)	46.4 (30.7-58.3)	
Pre-ACLF	7 (1%)	48.7 (36.1-54.7)	

Table S1. Comparison of NH3 levels according to different liver disease severity indicators.

Abbreviations: AMM-ULN ammonia level corrected to the upper limit of normal; *cACLD compensated advanced chronic liver disease; CS clinical stage; CTP Child-Turcotte-Pugh; dACLD decompensated advanced chronic liver disease; HVPG hepatic venous pressure gradient; UNOS MELD (2016) United Network for Organ Sharing model for end-stage liver disease (2016)*

Table S2

Patient characteristics	**Univariable**		**Model 1 (incl. CTP score, sodium, and creatinine)**		**Model 2 (incl. MELD, and albumin)**	
	**SHR (95%CI)**	**p-value**	**aSHR (95%CI)**	**p-value**	**aSHR (95%CI)**	**p-value**
Age, year	1.05 (1.03-1.08)	**<0.001**	1.06 (1.03-1.08)	**<0.001**	1.05 (1.03-1.08)	**<0.001**
HVPG, mmHg	1.07 (1.05-1.10)	**<0.001**	1.03 (0.99-1.07)	0.110	1.03 (0.99-1.07)	0.120
CTP score						
A	1		1		-	-
B	2.69 (1.75-4.13)	**<0.001**	1.64 (0.94-2.86)	0.085	-	-
C	4.67 (2.70-8.10)	**<0.001**	2.35 (1.04-5.33)	**0.040**	-	-
UNOS MELD (2016) score, point	1.09 (1.05-1.13)	**<0.001**	-	-	1.03 (0.98-1.08)	0.260
Decompensated vs. compensated ACLD	2.39 (1.59-3.58)	**<0.001**	-	-	0.98 (0.56-1.69)	0.940
Sodium, mmol x L^{-1}	0.93 (0.88-0.98)	**0.004**	0.99 (0.93-1.06)	0.850	-	-
Creatinine, mg x dL^{-1}	2.07 (1.16-3.70)	**0.014**	0.91 (0.45-1.82)	0.790	-	-
Albumin, g x L^{-1}	0.92 (0.90-0.95)	**<0.001**	-	-	0.97 (0.94-1.00)	0.078
CRP, mg x L^{-1}	2.02 (1.54-2.63)	**<0.001**	1.50 (1.09-2.06)	**0.014**	1.48 (1.05-2.07)	**0.024**
NH3-ULN ≥1.4 vs. <1.4	3.39 (2.08-5.53)	**<0.001**	2.19 (1.22-3.94)	**0.009**	2.41 (1.32-4.40)	**0.004**

Table S2. Competing risk regression analyses of factors associated with liver-related death including – among other parameters – CTP score, serum sodium, and creatinine **(model 1)** or UNOS MELD (2016) score, clinical stage, and serum albumin **(model 2)** with requirement of liver transplantation/non-liver-related death/removal of the primary etiological factor as competing risks in the **outcome cohort**.

Abbreviations: ACLD advanced chronic liver disease; aHR adjusted hazard ratio; NH3-ULN ammonia adjusted for the upper limit of normal; CI confidence interval; CRP C-reactive protein; CTP Child-Turcotte-Pugh score; HVPG hepatic venous pressure gradient; UNOS MELD (2016) United Network for Organ Sharing Model of End-stage Liver Disease (2016)

Table S3

Patient characteristics	**Univariable**		**Model 1 (incl. CTP score, sodium, and creatinine)**		**Model 2 (incl. MELD, and albumin)**	
	**HR (95%CI)**	**p-value**	**aHR (95%CI)**	**p-value**	**aHR (95%CI)**	**p-value**
Age, year	1.02 (1.01-1.03)	**0.008**	1.01 (0.99-1.02)	0.349	1.01 (0.99-1.02)	0.282
HVPG, mmHg	1.13 (1.11-1.16)	**<0.001**	1.10 (1.07-1.14)	**<0.001**	1.10 (1.07-1.14)	**<0.001**
CTP score						
A	1		1		-	-
B	3.92 (2.82-5.45)	**<0.001**	1.92 (1.30-2.82)	**<0.001**	-	-
C	4.56 (2.70-7.70)	**<0.001**	1.39 (0.73-2.66)	0.315	-	-
UNOS MELD (2016) score, point	1.10 (1.07-1.13)	**<0.001**	-	-	0.99 (0.95-1.03)	0.540
Decompensated vs. compensated ACLD	3.42 (2.46-4.75)	**<0.001**	-	-	1.47 (1.01-2.13)	**0.043**
Sodium, mmol x L^{-1}	0.91 (0.88-0.95)	**<0.001**	1.00 (0.95-1.04)	0.908	-	-
Creatinine, mg x dL^{-1}	1.73 (1.01-2.99)	**0.048**	1.18 (0.69-2.01)	0.550	-	-
Albumin, g x L^{-1}	0.90 (0.88-0.93)	**<0.001**	-	-	0.96 (0.93-0.99)	**0.020**
CRP, mg x L^{-1}	2.23 (1.76-2.81)	**<0.001**	1.63 (1.22-2.19)	**0.001**	1.61 (1.21-2.16)	**0.001**
NH3-ULN ≥1.4 vs. <1.4	3.57 (2.35-5.41)	**<0.001**	2.38 (1.51-3.73)	**<0.001**	2.19 (1.41-3.40)	**<0.001**
**Concordance ± SE**			**0.779 ± 0.019**		**0.775 ± 0.019**	
**AIC**			**1749.919**		**1751.330**	

Table S3. Uni- and multivariable Cox regression analyses of factors associated with hepatic decompensation including – among other parameters – CTP score, serum sodium, and creatinine (model 1) or UNOS MELD (2016) score, clinical stage, and serum albumin (model 2).

Abbreviations: ACLD advanced chronic liver disease; aHR adjusted hazard ratio; AIC Akaike information criterion; NH3-ULN ammonia adjusted for the upper limit of normal; CI confidence interval; CRP C-reactive protein; CTP Child-Turcotte-Pugh score; HVPG hepatic venous pressure gradient; UNOS MELD (2016) United Network for Organ Sharing Model of End-stage Liver Disease (2016)

Table S4

Patient characteristics	**Univariable**		**Model 1** (incl. CTP score, sodium, and creatinine)		**Model 2** (incl. MELD, and albumin)	
	**SHR (95%CI)**	**p-value**	**aSHR (95%CI)**	**p-value**	**aSHR (95%CI)**	**p-value**
Age, year	1.03 (1.02-1.05)	**<0.001**	1.02 (1.00-1.04)	**0.035**	1.02 (1.00-1.04)	**0.013**
HVPG, mmHg	1.11 (1.08-1.15)	**<0.001**	1.07 (1.04-1.11)	**<0.001**	1.06 (1.02-1.10)	**0.004**
CTP score						
A	1		1		-	-
B	4.49 (3.20-6.28)	**<0.001**	2.20 (1.44-3.34)	**<0.001**	-	-
C	6.17 (3.65-10.41)	**<0.001**	2.07 (1.00-4.27)	**0.049**	-	-
UNOS MELD (2016) score, point	1.11 (1.07-1.14)	**<0.001**	-	-	0.98 (0.94-1.03)	0.510
Decompensated vs. compensated ACLD	5.70 (3.97-8.17)	**<0.001**	-	-	2.74 (1.80-4.18)	**<0.001**
Sodium, mmol x L^{-1}	0.90 (0.86-0.93)	**<0.001**	0.97 (0.93-1.02)	0.300	-	-
Creatinine, mg x dL^{-1}	2.71 (1.66-4.41)	**<0.001**	1.56 (0.82-2.99)	0.180	-	-
Albumin, g x L^{-1}	0.89 (0.87-0.91)	**<0.001**	-	-	0.95 (0.91-0.98)	**0.002**
CRP, mg x L^{-1}	2.28 (1.75-2.96)	**<0.001**	1.51 (1.10-2.08)	**0.012**	1.49 (1.07-2.08)	**0.018**
NH3-ULN ≥1.4 vs. <1.4	4.11 (2.74-6.16)	**<0.001**	2.22 (1.27-3.90)	**0.005**	2.09 (1.23-3.55)	**0.006**

Table S4. Competing risk regression analyses of factors associated with hepatic decompensation/liver-related death including – among other parameters – CTP score, serum sodium, and creatinine **(model 1)** or UNOS MELD (2016) score, clinical stage, and serum albumin **(model 2)** with requirement of liver transplantation/non-liver-related death/removal of the primary etiological factor as competing risks in the **outcome cohort**.

Abbreviations: ACLD advanced chronic liver disease; aHR adjusted hazard ratio; NH3-ULN ammonia adjusted for the upper limit of normal; CI confidence interval; CRP C-reactive protein; CTP Child-Turcotte-Pugh score; HVPG hepatic venous pressure gradient; UNOS MELD (2016) United Network for Organ Sharing Model of End-stage Liver Disease (2016)

Table S5

Patient characteristics	**Univariable**		**Model 1 (incl. CTP score, sodium, and creatinine)**		**Model 2 (incl. MELD, and albumin)**	
	**HR (95%CI)**	**p-value**	**aHR (95%CI)**	**p-value**	**aHR (95%CI)**	**p-value**
Age, year	1.04 (1.03-1.06)	**<0.001**	1.04 (1.03-1.06)	**<0.001**	1.04 (1.02-1.05)	**<0.001**
HVPG, mmHg	1.10 (1.07-1.13)	**<0.001**	1.05 (1.02-1.08)	**0.001**	1.03 (1.00-1.07)	**0.046**
CTP score						
A	1		1		-	-
B	3.39 (2.41-4.77)	**<0.001**	2.07 (1.38-3.11)	**<0.001**	-	-
C	5.77 (3.57-9.34)	**<0.001**	3.45 (1.90-6.24)	**<0.001**	-	-
UNOS MELD (2016) score, point	1.11 (1.08-1.14)	**<0.001**	-	-	1.02 (0.98-1.06)	0.392
Decompensated vs. compensated ACLD	4.44 (3.11-6.34)	**<0.001**	-	-	2.37 (1.57-3.57)	**<0.001**
Sodium, mmol x L^{-1}	0.91 (0.88-0.94)	**<0.001**	0.97 (0.93-1.02)	0.226	-	-
Creatinine, mg x dL^{-1}	2.08 (1.22-3.55)	**0.007**	1.23 (0.71-2.13)	0.451	-	-
Albumin, g x L^{-1}	0.90 (0.88-0.92)	**<0.001**	-	-	0.94 (0.91-0.97)	**<0.001**
CRP, mg x L^{-1}	2.00 (1.58-2.53)	**<0.001**	1.20 (0.90-1.61)	0.221	1.16 (0.87-1.55)	0.326
NH3-ULN ≥1.4 vs. <1.4	3.12 (2.03-4.81)	**<0.001**	1.86 (1.17-2.95)	**0.008**	1.78 (1.14-2.79)	**0.011**
**Concordance ± SE**			**0.770 ± 0.020**		**0.788 ± 0.017**	
**AIC**			**1657.052**		**1639.358**	

Table S5. Uni- and multivariable Cox regression analyses of factors associated with non-elective liver-related hospitalisation/liver-related death including – among other parameters – CTP score, serum sodium, and creatinine **(model 1)** or UNOS MELD (2016) score, clinical stage, and serum albumin **(model 2)** in the **outcome cohort**.

Abbreviations: ACLD advanced chronic liver disease; aHR adjusted hazard ratio; AIC Akaike information criterion; AMM-ULN ammonia adjusted for the upper limit of normal; ARLD alcohol-related liver disease; CI confidence interval; CRP C-reactive protein; CTP Child-Turcotte-Pugh score; HVPG hepatic venous pressure gradient; NAFLD non-alcoholic fatty liver disease; SE standard error; UNOS MELD (2016) United Network for Organ Sharing Model of End-stage Liver Disease (2016)

Table S6

Patient characteristics	**Univariable**		**Model 1 (incl. CTP score, sodium, and creatinine)**		**Model 2 (incl. MELD, and albumin)**	
	**SHR (95%CI)**	**p-value**	**aSHR (95%CI)**	**p-value**	**aSHR (95%CI)**	**p-value**
Age, year	1.04 (1.02-1.06)	**<0.001**	1.04 (1.02-1.06)	**<0.001**	1.04 (1.02-1.05)	**<0.001**
HVPG, mmHg	1.07 (1.05-1.10)	**<0.001**	1.03 (0.99-1.06)	0.072	1.01 (0.98-1.05)	0.450
CTP score						
A	1		1		-	-
B	3.31 (2.32-4.73)	**<0.001**	2.08 (1.32-3.30)	**0.002**	-	-
C	5.62 (3.27-9.66)	**<0.001**	3.31 (1.67-6.57)	**<0.001**	-	-
UNOS MELD (2016) score, point	1.09 (1.06-1.13)	**<0.001**	-	-	1.01 (0.96-1.06)	0.840
Decompensated vs. compensated ACLD	4.45 (3.07-6.47)	**<0.001**	-	-	2.61 (1.61-4.22)	**<0.001**
Sodium, mmol x L^{-1}	0.91 (0.87-0.95)	**<0.001**	0.97 (0.92-1.02)	0.230	-	-
Creatinine, mg x dL^{-1}	2.40 (1.40-4.12)	**0.002**	1.35 (0.69-2.66)	0.390	-	-
Albumin, g x L^{-1}	0.91 (0.88-0.93)	**<0.001**	-	-	0.94 (0.91-0.98)	**<0.001**
CRP, mg x L^{-1}	1.92 (1.47-2.51)	**<0.001**	1.24 (0.91-1.69)	0.180	1.21 (0.87-1.68)	0.250
NH3-ULN ≥1.4 vs. <1.4	3.81 (2.46-5.90)	**<0.001**	2.06 (1.18-3.61)	**0.011**	2.12 (1.28-3.50)	**0.004**

Table S6. Competing risk regression analyses of factors associated with non-elective liver-related hospitalisation/liver-related death including – among other parameters – CTP-score, serum sodium, and creatinine **(model 1)** or UNOS MELD (2016) score, clinical stage, and serum albumin **(model 2)** with requirement of liver transplantation/non-liver-related death/removal of the primary etiological factor as competing risks in the **outcome cohort**.

Abbreviations: ACLD advanced chronic liver disease; aHR adjusted hazard ratio; NH3-ULN ammonia adjusted for the upper limit of normal; CI confidence interval; CRP C-reactive protein; CTP Child-Turcotte-Pugh score; HVPG hepatic venous pressure gradient; UNOS MELD (2016) United Network for Organ Sharing Model of End-stage Liver Disease (2016)

Table S7

Patient characteristics	<u>**Univariable**</u>		<u>**Model 1**</u> **(incl. CTP score, sodium, and creatinine)**		<u>**Model 2**</u> **(incl. MELD, and albumin)**	
	**HR (95%CI)**	**p-value**	**aHR (95%CI)**	**p-value**	**aHR (95%CI)**	**p-value**
Age, year	1.03 (1.01-1.05)	**0.012**	1.02 (1.00-1.04)	**0.036**	1.03 (1.01-1.05)	**0.007**
HVPG, mmHg	1.03 (0.99-1.07)	0.125	1.00 (0.96-1.04)	0.944	1.00 (0.96-1.04)	0.887
CTP score						
A	1		1		-	-
B	1.76 (1.07-2.89)	**0.026**	1.52 (0.89-2.60)	0.122	-	-
C	3.22 (1.79-5.79)	**<0.001**	2.93 (1.46-5.87)	**0.002**	-	-
UNOS MELD (2016) score, point	1.08 (1.04-1.12)	**<0.001**	-	-	1.07 (1.02-1.11)	**0.003**
Sodium, mmol x L^{-1}	0.96 (0.91-0.99)	**0.048**	0.99 (0.94-1.04)	0.549	-	-
Creatinine, mg x dL^{-1}	2.36 (1.34-4.14)	**0.003**	1.93 (1.02-3.65)	**0.045**	-	-
Albumin, g x L^{-1}	0.95 (0.92-0.97)	**<0.001**	-	-	0.97 (0.94-1.01)	0.147
CRP, mg x L^{-1}	1.36 (1.02-1.82)	**0.039**	1.08 (0.77-1.50)	0.668	1.13 (0.81-1.59)	0.473
NH3-ULN ≥1.4 vs. <1.4	1.91 (1.18-3.09)	**<0.001**	1.60 (0.96-2.65)	0.071	1.71 (1.05-2.80)	**0.031**
**Concordance ± SE**			**0.683 ± 0.029**		**0.689 ± 0.029**	
**AIC**			**954.323**		**949.870**	

Table S7. Uni- and multivariable Cox regression analyses of factors associated with the development of acute-on-chronic liver failure (ACLF)/requirement of liver transplantation/liver-related death including – among other parameters – CTP score, serum sodium, and creatinine **(<u>model 1</u>)** or UNOS MELD (2016) score and serum albumin **(<u>model 2</u>)** in patients who already have experienced decompensation at baseline in the **<u>outcome cohort</u>**.

Abbreviations: ACLD advanced chronic liver disease; aHR adjusted hazard ratio; AIC Akaike information criterion; NH3-ULN ammonia adjusted for the upper limit of normal; ARLD alcohol-related liver disease; CI confidence interval; CRP C-reactive protein; CTP Child-Turcotte-Pugh score; HVPG hepatic venous pressure gradient; NAFLD non-alcoholic fatty liver disease; SE standard error; UNOS MELD (2016) United Network for Organ Sharing Model of End-stage Liver Disease (2016)

Table S8

Patient characteristics	**Univariable**		**Model 1 (incl. CTP score, sodium, and creatinine)**		**Model 2 (incl. MELD, and albumin)**	
	**SHR (95%CI)**	**p-value**	**aSHR (95%CI)**	**p-value**	**aSHR (95%CI)**	**p-value**
Age, year	1.02 (1.00-1.04)	**0.022**	1.02 (1.00-1.04)	0.051	1.03 (1.01-1.05)	**0.015**
HVPG, mmHg	1.02 (0.98-1.05)	0.360	0.99 (0.95-1.03)	0.560	0.98 (0.94-1.02)	0.410
CTP score						
A	1		1		-	-
B	1.65 (0.99-2.74)	0.053	1.42 (0.82-2.46)	0.220	-	-
C	3.28 (1.82-5.91)	**<0.001**	2.92 (1.42-6.02)	**0.004**	-	-
UNOS MELD (2016) score, point	1.09 (1.05-1.13)	**<0.001**	-	-	1.08 (1.03-1.13)	**0.003**
Sodium, mmol x L^{-1}	0.94 (0.89-0.99)	**0.032**	0.97 (0.91-1.03)	0.270	-	-
Creatinine, mg x dL^{-1}	2.36 (1.24-4.50)	**0.009**	2.03 (0.93-4.46)	0.077	-	-
Albumin, g x L^{-1}	0.95 (0.92-0.98)	**<0.001**	-	-	0.98 (0.94-1.02)	0.240
CRP, mg x L^{-1}	1.38 (1.02-1.87)	**0.035**	1.11 (0.78-1.57)	0.560	1.21 (0.86-1.71)	0.270
NH3-ULN ≥1.4 vs. <1.4	2.16 (1.36-3.45)	**0.001**	1.80 (1.06-3.06)	**0.030**	1.93 (1.16-3.21)	**0.011**

Table S8. Competing risk regression analyses of factors associated with the development of ACLF/requirement of liver transplantation/liver-related death including – among other parameters – CTP-score, serum sodium, and creatinine **(model 1)** or serum albumin, and UNOS MELD (2016)-score **(model 2)** with non-liver-related death/removal of the primary etiological factor as competing risks in patients with who have experienced decompensation prior to study inclusion in the **outcome cohort**.

Abbreviations: ACLD advanced chronic liver disease; ACLF acute-on-chronic liver failure; aHR adjusted hazard ratio; NH3-ULN ammonia adjusted for the upper limit of normal; CI confidence interval; CRP C-reactive protein; CTP Child-Turcotte-Pugh score; HVPG hepatic venous pressure gradient; UNOS MELD (2016) United Network for Organ Sharing Model of End-stage Liver Disease (2016)
