# Supplementary material for: Impact of ammonia levels on outcome in clinically stable outpatients with advanced chronic liver disease
Source: JHEP Rep. 2023 Jan 23;5(4):100682. doi: 10.1016/j.jhepr.2023.100682 (PMC9976454; doi:10.1016/j.jhepr.2023.100682)
Supplement: Multimedia component 4 [file mmc4.pdf]

Research article

JHEP Reports

Impact of ammonia levels on outcome in clinically stable outpatients with advanced chronic liver disease

Lorenz Balcar,[1,2,†] **Julia Krawanja**,[1,2,†] Bernhard Scheiner,[1,2] Rafael Paternostro,[1,2] Benedikt Simbrunner,[1,2] Georg Semmler,[1,2] Mathias Jachs,[1,2] Lukas Hartl,[1,2] Albert Friedrich Stättermayer,[1,2] Philipp Schwabl,[1,2] Matthias Pinter,[1] Thomas Szekeres,[3] Michael Trauner,[1] Thomas Reiberger,[1,2,‡] Mattias Mandorfer[1,2,*,‡]

[1]*Division of Gastroenterology and Hepatology, Department of Internal Medicine III, Medical University of Vienna, Vienna, Austria;* [2]*Vienna Hepatic Hemodynamic Lab, Division of Gastroenterology and Hepatology, Department of Internal Medicine III, Medical University of Vienna, Vienna, Austria;* [3]*Department of Laboratory Medicine, Medical University of Vienna, Vienna, Austria*

JHEP Reports **2023.** https://doi.org/10.1016/j.jhepr.2023.100682

Background & Aims: Ammonia levels predicted hospitalisation in a recent landmark study not accounting for portal hypertension and systemic inflammation severity. We investigated (i) the prognostic value of venous ammonia levels (outcome cohort) for liver-related outcomes while accounting for these factors and (ii) its correlation with key disease-driving mechanisms (biomarker cohort).
Methods: (i) The outcome cohort included 549 clinically stable outpatients with evidence of advanced chronic liver disease. (ii) The partly overlapping biomarker cohort comprised 193 individuals, recruited from the prospective Vienna Cirrhosis Study (VICIS: NCT03267615).
Results: (i) In the outcome cohort, ammonia increased across clinical stages as well as hepatic venous pressure gradient and United Network for Organ Sharing model for end-stage liver disease (2016) strata and were independently linked with diabetes. Ammonia was associated with liver-related death, even after multivariable adjustment (adjusted hazard ratio [aHR]: 1.05 [95% CI: 1.00–1.10]; $p = 0.044$). The recently proposed cut-off (≥1.4 × upper limit of normal) was independently predictive of hepatic decompensation (aHR: 2.08 [95% CI: 1.35–3.22]; $p < 0.001$), non-elective liver-related hospitalisation (aHR: 1.86 [95% CI: 1.17–2.95]; $p = 0.008$), and – in those with decompensated advanced chronic liver disease – acute-on-chronic liver failure (aHR: 1.71 [95% CI: 1.05–2.80]; $p = 0.031$). (ii) Besides hepatic venous pressure gradient, venous ammonia was correlated with markers of endothelial dysfunction and liver fibrogenesis/matrix remodelling in the biomarker cohort.
Conclusions: Venous ammonia predicts hepatic decompensation, non-elective liver-related hospitalisation, acute-on-chronic liver failure, and liver-related death, independently of established prognostic indicators including C-reactive protein and hepatic venous pressure gradient. Although venous ammonia is linked with several key disease-driving mechanisms, its prognostic value is not explained by associated hepatic dysfunction, systemic inflammation, or portal hypertension severity, suggesting direct toxicity.
Impact and implications: A recent landmark study linked ammonia levels (a simple blood test) with hospitalisation/death in individuals with clinically stable cirrhosis. Our study extends the prognostic value of venous ammonia to other important liver-related complications. Although venous ammonia is linked with several key disease-driving mechanisms, they do not fully explain its prognostic value. This supports the concept of direct ammonia toxicity and ammonia-lowering drugs as disease-modifying treatment.
© 2023 The Authors. Published by Elsevier B.V. on behalf of European Association for the Study of the Liver (EASL). This is an open access article under the CC BY license (http://creativecommons.org/licenses/by/4.0/).

Introduction

Advanced chronic liver disease (ACLD) is a major source of morbidity and mortality worldwide, with non-alcoholic fatty liver disease (NAFLD) emerging as the predominant cause of ACLD in several regions.[1,2] The first development of hepatic decompensation – most commonly ascites, although hepatic encephalopathy (HE) is the predominant first event in NAFLD[2] – denotes a watershed moment in the natural history of ACLD, as it is accompanied by a substantial increase in long-term mortality.[3] Those with decompensated ACLD (dACLD) are at risk of acute-on-chronic liver failure (ACLF), which is defined by extrahepatic organ dysfunction (including acute encephalopathy) and

Keywords: Hepatic encephalopathy; Cirrhosis; Decompensation; Death; Acute-on-chronic liver failure.
Received 18 October 2022; received in revised form 28 December 2022; accepted 10 January 2023; available online 23 January 2023
† These authors share first authorship.
‡ These authors share senior authorship.
* Corresponding author. Address: Division of Gastroenterology and Hepatology, Department of Internal Medicine III, Medical University of Vienna, Waehringer Guertel 18-20, 1090 Vienna, Austria. Tel.: +43 1 40400 47440; fax: +43 1 40400 47350.
E-mail address: mattias.mandorfer@meduniwien.ac.at (M. Mandorfer).

ELSEVIER

EASL The Home of Hepatology

high short-term mortality.[4] Portal hypertension, which is accompanied by portosystemic shunting, and bacterial translocation-induced systemic inflammation are considered as the main drivers of clinical deterioration.[5]

In people with ACLD, hyperammonaemia is driven by microbiome changes and ammonia overproduction,[6,7] decreased hepatic/extrahepatic metabolic capacity (urea cycle and glutamine synthetase[8,9]), and portosystemic shunting via collaterals.[10,11] The diagnostic utility of ammonia testing for HE is controversially discussed, as hyperammonaemia is not the only mechanism for HE development, and thus, people with HE may present with normal ammonia levels.[10] Nevertheless, in those with altered mental status, values within the normal range may question the diagnosis of HE.[12]

Notably, experimental studies indicate ammonia toxicity beyond its role in HE (*e.g.* liver fibrogenesis and immune dysfunction[13,14]). In a recent landmark study, Tranah *et al.*[15] evaluated the impact of ammonia on liver-related outcomes in clinically stable individuals with ACLD and values ≥1.4 × the upper limit of normal predicted liver-related events in stable outpatients with ACLD. However, it remains unclear whether this association is independent of portal hypertension/systemic inflammation, that is, well-established disease-driving mechanisms. Moreover, the findings of experimental studies, that is the link between hyperammonaemia and liver fibrogenesis, remains to be confirmed in humans to support the potential role of ammonia-lowering drugs as a disease-modifying treatment.

The objectives of our study were (i) to externally validate and extend previous findings on the prognostic value of venous plasma ammonia levels in a large, well-characterised cohort, while accounting for portal hypertension and systemic inflammation severity and (ii) to investigate the relationship between venous plasma ammonia and biomarkers of other disease-driving mechanisms.

Patients and methods

Study design and participants

We performed a retrospective, single-centre cohort study in individuals with ACLD who underwent hepatic venous pressure gradient (HVPG) measurement at the Vienna Hepatic Hemodynamic Lab (outcome cohort; Fig. S1). Those from the outcome cohort were included between Q2/04 and Q4/20. Inclusion criteria were (i) liver stiffness measurement ≥10 kPa and/or HVPG ≥6 mmHg, and (ii) availability of venous plasma ammonia levels. Furthermore, individuals were excluded if any of the following criteria were present: those with a history of orthotopic liver transplantation, any active extrahepatic malignancy, non-parenchymal liver disease, non-elective hospitalisation as a result of a liver-related complication at HVPG measurement or within 28 days before HVPG measurement, unsuccessful/unreliable HVPG measurement, bacterial infection, or missing information on important laboratory parameters and/or clinical follow-up. Recruitment and follow-up over the study period are depicted in Fig. S2.

In addition, we assessed biomarkers in a partly overlapping cohort of individuals (n = 193) from the prospective Vienna Cirrhosis Study (VICIS; NCT03267615; pathophysiology cohort) who were recruited between Q1/2017 and Q3/2022 (Fig. S1), applying similar inclusion and exclusion criteria (biomarker cohort). Overall, n = 82 participants are included in both cohorts (15% [82/549] *vs.* 42% [82/193]).

HVPG measurement

Under local anaesthesia and ultrasound guidance, a catheter introducer sheath was inserted into the right internal jugular vein.[16] Subsequently, a hepatic vein was cannulated and the free and wedged hepatic venous pressures were obtained at least as triplicate measurements by a balloon catheter,[17] as recommended by Baveno VII.[18]

Measurement of biomarkers

Routine laboratory tests, venous plasma ammonia, and biomarkers (von Willebrand factor [vWF], procalcitonin [PCT], IL-6, enhanced liver fibrosis [ELF®] test, copeptin, renin, and bile acids [BAs]) were performed by the ISO-certified Department of Laboratory Medicine of the Medical University of Vienna using commercially available methods that are applied in clinical routine and blood samples obtained via a central venous line (*i.e.* the side port of the catheter introducer sheath) at the time of HVPG measurement. Venous plasma ammonia was sampled and rapidly transported on ice to the central laboratory. In line with the previous landmark study,[15] venous plasma ammonia levels were divided by the sex-specific upper limit of normal of our local laboratory (*i.e.* 60 mmol/L for males and 51 mmol/L for females).

Clinical stages of ACLD, definition of hepatic decompensation and of ACLF

Participants were classified according to recently defined prognostic/clinical stages. The definition was adapted from D'Amico *et al.*[19] dACLD was defined by the presence or history of at least one decompensating event, that is ascites, variceal bleeding, or HE. ACLF was defined according to European Foundation for the Study of Chronic Liver Failure (EF-CLIF) criteria.[20]

Statistical analysis

All statistical analyses were performed using IBM SPSS Statistics 27 (IBM, New York, NY, USA), R 4.1.2 (R Core Team, R Foundation for Statistical Computing, Vienna, Austria), or GraphPad Prism 8 (GraphPad Software, San Diego, CA, USA). Categorical variables were reported as absolute (n) and relative frequencies (%), whereas continuous variables as mean ± SD or median (interquartile range [IQR]), as appropriate. Student's *t* test was used for group comparisons of normally distributed variables and the Mann–Whitney *U* test for non-normally distributed variables. Group comparisons of categorical variables were performed using either X^2 or Fisher's exact test, as appropriate.

Univariable and multivariable linear regression analyses were applied to evaluate factors associated with ammonia.

Follow-up time was calculated as the time from HVPG measurement to the date of liver transplantation, death, or last follow-up at one of the hospitals of the Vienna hospital association by the reverse Kaplan–Meier method. Impact of venous plasma ammonia levels on liver-related outcomes was assessed using Cox regression and competing risk analyses considering the removal/suppression of the primary aetiological factor (as defined by Baveno VII,[18] *i.e.* initiation of antiviral therapy/reported alcohol abstinence), liver transplantation, or non-liver-related death, as competing risks. Analyses were performed for hepatic decompensation/liver-related death, liver-related death, development of ACLF/requirement of liver transplantation/liver-related death, and non-elective liver-related hospitalisation/liver-related death as outcomes of interest. For the outcome development of ACLF/requirement of liver transplantation/liver-related death – in

Table 1. Detailed patient characteristics at the time of HVPG measurement of the outcome and the pathophysiology cohort.

Patient characteristics	Outcome cohort, n = 549	Pathophysiology cohort, n = 193	*p* value
Age, years, mean ± SD	54.4 ± 11.5	55.6 ± 12.4	0.208
Sex, n (%)			
Male	370 (67%)	131 (68%)	0.902
Female	179 (33%)	62 (32%)	
Aetiology, n (%)			
Viral	207 (38%)	36 (19%)	**<0.001**
ARLD	196 (36%)	82 (43%)	
NAFLD	57 (10%)	20 (10%)	
Other	89 (16%)	55 (29%)	
Varices, n (%)	319 (65%)	106 (65%)	1.000
History of decompensation, n (%)	252 (46%)	119 (62%)	**<0.001**
History of variceal bleeding, n (%)	67 (12%)	21 (11%)	0.625
Ascites, n (%)			
None	354 (65%)	105 (54%)	**0.036**
Mild	160 (29%)	75 (39%)	
Severe	35 (6%)	13 (7%)	
History of hepatic encephalopathy, n (%)	42 (8%)	30 (16%)	0.545
HVPG, mmHg, mean ± SD	16 ± 7	15 ± 6	0.190
HVPG 0–5 mmHg, n (%)	42 (8%)	–	**<0.001**
HVPG 6–9 mmHg, n (%)	83 (15%)	44 (23%)	
HVPG 10-15 mmHg, n (%)	137 (25%)	61 (32%)	
HVPG ≥16 mmHg, n (%)	287 (52%)	88 (46%)	
UNOS MELD (2016), points, mean ± SD	11.8 ± 4.5	11.4 ± 4.6	0.326
CTP score, points, mean ± SD	6.5 ± 1.7	6.6 ± 1.9	0.541
A, n (%)	342 (62%)	117 (61%)	0.475
B, n (%)	168 (31%)	57 (30%)	
C, n (%)	39 (7%)	19 (9%)	
Laboratory parameters, median (IQR) or mean ± SD			
Platelet count, G/L	107 (73–152)	103 (70–142)	0.248
Sodium, mmol/L	138.0 ± 3.7	138.1 ± 3.7	0.788
Creatinine, mg/dl	0.7 (0.6–0.9)	0.8 (0.6–0.9)	0.401
Albumin, g/L	36.5 ± 5.7	37.2 ± 5.5	0.136
Bilirubin, mg/dl	1.0 (0.7–1.9)	1.0 (0.6–1.8)	0.638
INR	1.3 ± 0.3	1.4 ± 0.3	0.438
AST, U/L	48 (34–67)	40 (29–55)	**<0.001**
ALT, U/L	35 (23–60)	30 (22–42)	**<0.001**
CRP, mg/L	0.2 (0.1–0.6)	0.2 (0.1–0.5)	0.730
Ammonia, mmol/L	37.3 (28.2–51.6)	34.5 (26.0–47.4)	0.061
NH_3-ULN	0.66 (0.49–0.91)	0.58 (0.43–0.79)	0.061

Categorical variables were reported as absolute (n) and relative frequencies (%), whereas continuous variables as mean ± SD or median (IQR), as appropriate. Student's *t* test was used for group comparisons of normally distributed variables and Mann–Whitney *U* test for non-normally distributed variables. Group comparisons of categorical variables were performed using either Chi-squared or Fisher's exact test, as appropriate. Values of *p* in bold denote *p* <0.05. ALT, alanine transaminase; ARLD, alcohol-related liver disease; AST aspartate transaminase; CRP, C-reactive protein; CTP, Child–Turcotte–Pugh; HVPG, hepatic venous pressure gradient; INR, international normalised ratio; NAFLD, non-alcoholic fatty liver disease; NH_3-ULN, ammonia level corrected to the upper limit of normal; UNOS MELD (2016) score, United Network for Organ Sharing model for end-stage liver disease (2016).

individuals who had already experienced hepatic decompensation at baseline (*i.e.* the main at-risk population) – removal/suppression of the primary aetiological factor or non-liver-related death were considered as competing risks. For competing risk regression analyses, Fine and Gray competing risks regression models (cmprsk: subdistribution analysis of competing risks, https://CRAN.R-project.org/package=cmprsk)[21,22] were calculated. Univariable and multivariable Cox regression analyses were performed to evaluate parameters independently associated with the events of interest. In a first step, we included all parameters into univariable Cox regression models. Baseline characteristics which we considered of particular importance for the endpoint of interest (*i.e.* age, indicators of hepatic dysfunction, HVPG, and C-reactive protein [CRP]) were further included into two separate multivariable models. The Child–Turcotte–Pugh (CTP) and United Network for Organ Sharing (UNOS) model for end-stage liver disease (MELD) (2016) scores have significant overlap in terms of included variables. Therefore, we generated separate models with either CTP or UNOS MELD (2016) scores.

Time-dependent area under the receiver operating characteristic curve (AUROC) analyses were performed and the R-package 'timeROC' was used to compare the prognostic performances for hepatic decompensation/liver-related death and liver-related death between established prognostic indicators (UNOS MELD [2016] score and HVPG) and ammonia (multiplicity-adjusted *p* values) over time.

Spearman's correlation analyses were conducted to investigate potential associations between ammonia and biomarkers in the biomarker cohort. A heatmap plot was used for graphical illustration of associations between ammonia and biomarkers.

The level of significance was set at a two-sided *p* value of <0.05.

Ethics

This study has been conducted in accordance with the principles of the Declaration of Helsinki and its amendments and has been approved by the local ethics committee (EK1531/2022 and EK1262/2017), which waived the requirement of written

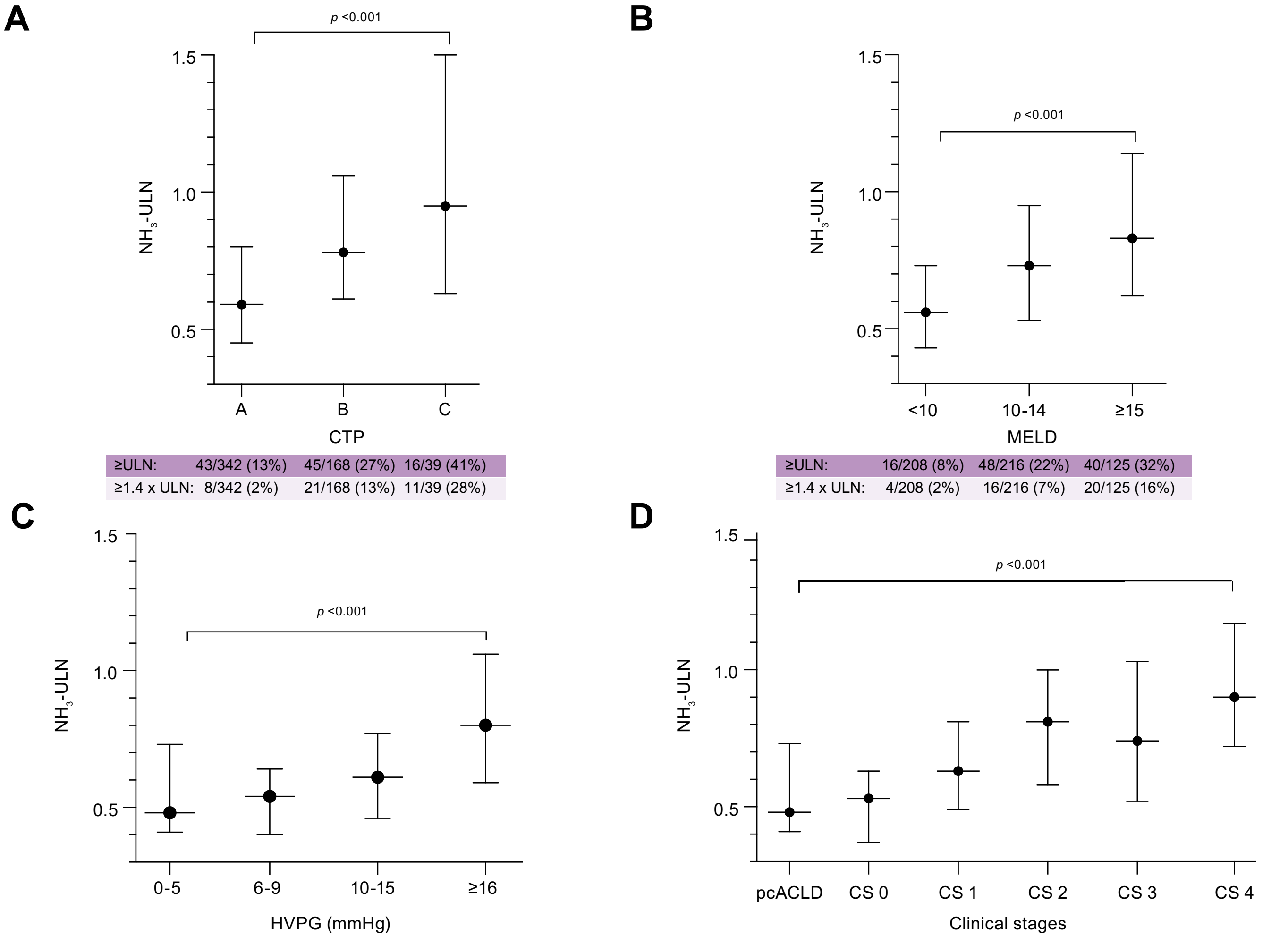

	A	B	C
≥ULN:	43/342 (13%)	45/168 (27%)	16/39 (41%)
≥1.4 x ULN:	8/342 (2%)	21/168 (13%)	11/39 (28%)

	<10	10-14	≥15
≥ULN:	16/208 (8%)	48/216 (22%)	40/125 (32%)
≥1.4 x ULN:	4/208 (2%)	16/216 (7%)	20/125 (16%)

	0-5	6-9	10-15	≥16
≥ULN:	3/42 (7%)	6/83 (7%)	14/137 (10%)	81/287 (28%)
≥1.4 x ULN:	2/42 (5%)	2/83 (2%)	9/137 (7%)	27/287 (9%)

	pcACLD	CS 0	CS 1	CS 2	CS 3	CS 4
≥ULN:	3/42 (7%)	6/74 (8%)	23/185 (12%)	6/25 (24%)	35/132 (27%)	31/91 (34%)
≥1.4 x ULN:	2/42 (5%)	2/74 (3%)	4/185 (2%)	3/25 (12%)	11/132 (8%)	18/91 (20%)

Fig. 1. NH_3-ULN across liver disease severity. Comparison of NH_3 corrected to the upper limit of normal according to (A) CTP (B) UNOS MELD (2016) score and (C) HVPG strata as well as (D) clinical stages in the outcome cohort. NH_3-ULN levels were reported as median (IQR) and compared with the Mann–Whitney *U* test. CS, clinical stages; CTP, CTP, Child–Turcotte–Pugh score; HVPG, hepatic venous pressure gradient; NH_3-ULN, ammonia adjusted for the upper limit of normal; pc, probably compensated; ULN, upper limit of normal; UNOS MELD (2016) score, United Network for Organ Sharing Model of end-stage liver disease (2016) score.

informed consent for the retrospective analysis of the outcome cohort. All participants included in the prospective biomarker cohort (*i.e.* VICIS study) provided written informed consent for study participation.

Results

Study population of the outcome cohort

Overall, 2,550 individuals underwent HVPG measurement within the study period (Fig. S1). After applying inclusion and exclusion criteria, 549 people were finally included into the outcome cohort. Mean age at HVPG measurement was 54 ± 12 years and most were male (n = 370, 67%; Table 1). Viral hepatitis was the most common aetiology of liver disease (n = 207, 38%), followed by alcohol-related liver disease (ARLD; n = 196, 36%), other aetiologies of ACLD (n = 89, 16%) and NAFLD (n = 57, 10%). Regarding portal hypertension severity, mean HVPG was 16 ± 7 mmHg and 65% of individuals (n = 319) had varices, of whom 67 (12%) had a history of variceal bleeding. Mean UNOS MELD (2016) was 12 ± 5, and mean CTP score was 7 ± 2 points. Most individuals were classified as CTP-A (n = 342, 62%), whereas 31% of participants were classified as CTP-B (n = 168) and 7% as CTP-C (n = 39). A total of 252 participants (46%) had already experienced hepatic decompensation at study inclusion. Eight percent (n = 42) had a history of overt HE. Accordingly, 7% (n = 38) were on lactulose, 5% (n = 27) on rifaximin, and 10% (n = 54) on oral L-ornithine *O*-aspartate at baseline. The median baseline ammonia level was 37.3 (IQR: 28.2–51.6) mmol/L and ammonia adjusted for the upper limit of normal (NH_3-ULN) was 0.66 (IQR: 0.49–0.91).

Clinical events during follow-up in the outcome cohort

Participants were followed for a median of 41.0 (95% CI: 37.3–44.7) months. A total of 104 deaths (19%) were considered liver-related, whereas 175 events (32%) were captured for the combined endpoint hepatic decompensation/liver-related death. For the endpoint liver-related death, 199 competing risks (36%)

Table 2. Simple and multiple linear regression analysis of factors associated with NH_3 corrected for the upper limit of normal including – among other parameters – either CTP score, as well as serum sodium and creatinine (model 1), or UNOS MELD (2016) score, clinical stage, and serum albumin (model 2) in the outcome cohort.

Patient characteristics	Univariable			Model 1 (including CTP score, sodium, creatinine)			Model 2 (including MELD and albumin)		
	B	95% CI	*p* value	B	95% CI	*p* value	B	95% CI	*p* value
Age, year	0.002	-0.002, 0.006	0.250	–	–	–	–	–	–
Male sex	-0.142	-0.232, -0.052	**0.002**	-0.174	-0.285, -0.064	**0.002**	-0.164	-0.272, -0.055	**0.003**
BMI, kg/m^2	0.012	0.004, 0.020	**0.005**	0.013	0.002, 0.023	**0.019**	0.014	0.003, 0.024	**0.010**
Overweight[†]	0.085	0.000, 0.171	**0.049**	–	–	–	–	–	–
Obesity[‡]	0.080	-0.021, 0.181	0.121	–	–	–	–	–	–
Prediabetes[§]	-0.012	-0.127, 0.103	0.839	–	–	–	–	–	–
Diabetes[#]	0.130	0.016, 0.243	**0.025**	0.123	0.004, 0.241	**0.043**	0.134	0.016, 0.252	**0.026**
Arterial hypertension[¶]	-0.099	-0.185, -0.013	**0.024**	-0.092	-0.202, 0.018	0.099	-0.073	-0.185, 0.038	0.197
Hypertriglyceridemia[††]	-0.099	-0.244, 0.047	0.184	–	–	–	–	–	–
Hypercholesterolemia[‡‡]	0.044	-0.091, 0.179	0.524	–	–	–	–	–	–
HDL below threshold[§§]	-0.016	-0.111, 0.079	0.744	–	–	–	–	–	–
Statin use	-0.100	-0.251, 0.051	0.192	–	–	–	–	–	–
Hepatic steatosis[##]	0.064	-0.030, 0.158	0.183	–	–	–	–	–	–
ARLD *vs.* other aetiologies	0.074	-0.015, 0.163	0.102	–	–	–	–	–	–
Varices	0.128	0.073, 0.183	**<0.001**	0.087	0.020, 0.154	**0.011**	0.069	0.001, 0.138	**0.048**
CTP score, point	0.095	0.070, 0.117	**<0.001**	0.087	0.050, 0.124	**<0.001**	–	–	–
UNOS MELD (2016), point	0.032	0.023, 0.041	**<0.001**	–	–	–	0.022	0.008, 0.036	**0.002**
HVPG, mmHg	0.020	0.014, 0.026	**<0.001**	0.006	-0.004, 0.015	0.234	0.004	-0.006, 0.013	0.417
Decompensated *vs.* compensated ACLD	0.276	0.194, 0.358	**<0.001**	–	–	–	0.117	0.001, 0.234	**0.048**
Sodium, mmol/L	-0.017	-0.028, -0.005	**0.004**	0.009	-0.007, 0.024	0.266	–	–	–
Creatinine, mg/dl	0.107	-0.054, 0.268	0.192	0.231	0.032, 0.430	**0.023**	–	–	–
Albumin, g/L	-0.021	-0.029, -0.014	**<0.001**	–	–	–	-0.004	-0.015, 0.007	0.519
ALT, U/L	0.000	0.000, 0.000	0.314	–	–	–	–	–	–
CRP, mg/L	0.109	0.026, 0.192	**0.011**	-0.037	-0.150, 0.077	0.526	-0.020	-0.133, 0.093	0.727

Values of *p* in bold denote *p* <0.05.
[†] BMI ≥25 kg/m^2.
[‡] BMI ≥30 kg/m^2.
[§] Fasting blood glucose 100–125 mg/dl; HbA1c 5.7–6.4%.
[#] Fasting blood glucose >125 mg/dl, HbA1c ≥6.5%, or antidiabetic medication.
[¶] Blood pressure >140/90 mmHg, or antihypertensive medication.
[††] Triglycerides >150 mg/dl.
[‡‡] Total cholesterol >200 mg/dl.
[§§] Value <35 mg/dl for males and <39 mg/dl for females.
[##] Biopsy-proven, controlled attenuation parameter >248 dB/m, or diagnosed by ultrasound. ALT alanine transaminase; ARLD alcohol-related liver disease; CRP, C-reactive protein; CTP, Child–Turcotte–Pugh score; HVPG, hepatic venous pressure gradient; UNOS MELD (2016) score, United Network for Organ Sharing model for end-stage liver disease (2016) score.

occurred, whereas for the combined endpoint (hepatic decompensation/liver-related death), 176 competing risks (32%) were captured. Among decompensated individuals, 45 (18%) developed ACLF.

Ammonia levels increase with liver disease and portal hypertension severity in the outcome cohort

NH_3-ULN/NH_3 consistently increased with liver disease/portal hypertension severity, as evaluated by the CTP score (*p* <0.001) and UNOS MELD (2016) score (*p* <0.001), as well as severity of portal hypertension (*p* <0.001; Fig. 1; Table S1). Additionally, NH_3-ULN also increased across clinical stages (*p* <0.001).

Univariable and multivariable analyses of factors associated with ammonia in the outcome cohort

In univariable analyses, NH_3-ULN was directly associated with severity of liver disease (CTP score: unstandardised regression coefficient [B]: 0.095 [95% CI: 0.070, 0.117]; *p* <0.001, UNOS MELD [2016] score: B: 0.032 [95% CI: 0.023, 0.041]; *p* <0.001), and portal hypertension severity (HVPG: B: 0.020 [95% CI: 0.014, 0.026]; *p* <0.001; Table 2). Additionally, there was a positive association with systemic inflammation (CRP: B: 0.109 [95% CI: 0.026, 0.192]; *p* = 0.011), and with body mass index (BMI: B: 0.012 [95% CI: 0.004, 0.020]; *p* = 0.005), presence of varices (B: 0.128 [95% CI: 0.073, 0.183]; *p* <0.001), diabetes (B: 0.130 [95% CI: 0.016, 0.243]; *p* = 0.025), and dACLD (B: 0.276 [95% CI: 0.194, 0.358]; *p* <0.001). Finally, NH_3-ULN was negatively associated with male sex (B: -0.142 [95% CI: -0.232, -0.052]; *p* = 0.002), arterial hypertension (B: -0.099 [95% CI: -0.185, -0.013]; *p* = 0.024), as well as serum sodium (B: -0.017 [95% CI: -0.028, -0.005]; *p* = 0.004) and albumin levels (B: -0.021 [95% CI: -0.029, -0.014]; *p* <0.001).

After multivariable adjustment for either CTP score, sodium, and creatinine (model 1) or UNOS MELD (2016) score, serum albumin, and dACLD (model 2), severity of liver disease (model 1: CTP score: B: 0.087 [95% CI: 0.050, 0.124]; *p* <0.001; model 2: UNOS MELD [2016] score: B: 0.022 [95% CI: 0.008, 0.036]; *p* = 0.002), presence of dACLD (model 2: B: 0.117 [95% CI: 0.001, 0.234]; *p* = 0.048), BMI (model 1: B: 0.013 [95% CI: 0.002, 0.023]; *p* = 0.019; model 2: B: 0.014 [95% CI: 0.003, 0.024]; *p* = 0.010), male sex (model 1: B: -0.174 [95% CI: -0.285, -0.064]; *p* = 0.002; model 2: B: -0.164 [95% CI: -0.272, -0.055]; *p* = 0.003), diabetes (model 1: B: 0.123 [95% CI: 0.004, 0.241]; *p* = 0.043; model 2: B: 0.134 [95% CI: 0.016, 0.252]; *p* = 0.026), presence of varices (model 1: B: 0.087 [95% CI: 0.020, 0.154]; *p* = 0.011; model 2: B: 0.069 [95% CI: 0.001, 0.138]; *p* = 0.048), and creatinine levels (model 1: B: 0.231 [95% CI: 0.032, 0.430]; *p* = 0.023) were the only parameters with independent positive associations (Table 2).

Table 3. Uni- and multivariable Cox regression analyses of factors associated with liver-related death including – among other parameters – CTP score, serum sodium, and creatinine (model 1) or UNOS MELD (2016) score, clinical stage, and serum albumin (model 2) in the outcome cohort.

Patient characteristics	Univariable		Model 1 (including CTP score, sodium, and creatinine)		Model 2 (including MELD and albumin)	
	HR (95% CI)	*p* value	aHR (95% CI)	*p* value	aHR (95% CI)	*p* value
Age, year	1.05 (1.03–1.07)	**<0.001**	1.05 (1.03–1.08)	**<0.001**	1.05 (1.03–1.07)	**<0.001**
HVPG, mmHg	1.09 (1.06–1.13)	**<0.001**	1.03 (0.99–1.07)	0.124	1.03 (0.99–1.07)	0.119
CTP score						
A	1		1		–	–
B	3.05 (1.99–4.69)	**<0.001**	2.04 (1.23–3.38)	**0.006**	–	–
C	5.89 (3.40–10.21)	**<0.001**	3.55 (1.76–7.17)	**<0.001**	–	–
UNOS MELD (2016) score, point	1.11 (1.07–1.15)	**<0.001**	–	–	1.04 (0.99–1.09)	0.080
Decompensated *vs.* compensated ACLD	2.38 (1.60–3.54)	**<0.001**	–	–	1.02 (0.63–1.66)	0.934
Sodium, mmol/L	0.92 (0.88–0.96)	**<0.001**	0.99 (0.94–1.05)	0.812	–	–
Creatinine, mg/dl	1.91 (0.99–3.71)	0.055	0.72 (0.35–1.49)	0.371	–	–
Albumin, g/L	0.92 (0.89–0.94)	**<0.001**	–	–	0.95 (0.92–0.99)	**0.003**
CRP, mg/L	2.08 (1.59–2.71)	**<0.001**	1.36 (0.98–1.90)	0.070	1.25 (0.87–1.78)	0.227
NH_3, µmol/L, per 10	1.08 (1.05–1.12)	**<0.001**	1.05 (1.00–1.10)	**0.044**	1.04 (1.00–1.08)	**0.049**
Concordance ± SE				**0.764 ± 0.024**		**0.776 ± 0.023**
AIC				**1,084.940**		**1,084.510**

Values of *p* in bold denote *p* <0.05. ACLD, advanced chronic liver disease; aHR, adjusted hazard ratio; AIC, Akaike information criterion; ARLD, alcohol-related liver disease; CRP, C-reactive protein; CTP, Child–Turcotte–Pugh score; HVPG, hepatic venous pressure gradient; NAFLD, non-alcoholic fatty liver disease; NH_3-ULN ammonia adjusted for the upper limit of normal; UNOS MELD (2016) score, United Network for Organ Sharing model of end-stage liver disease (2016) score.

Impact of ammonia on liver-related outcomes in the outcome cohort

NH_3 not only increased with liver disease severity in cross-sectional analyses but was also longitudinally associated with liver-related death (HR: 1.08 95% CI: 1.05–1.12]; *p* <0.001). Its independent prognostic value was confirmed in two multivariable models (model 1: adjusted HR [aHR]: 1.05 [95% CI: 1.00–1.12]; *p* = 0.044; model 2: aHR: 1.04 [95% CI: 1.00–1.08]; *p* = 0.049), which were adjusted for age, HVPG, and CRP as well as additionally CTP-score, sodium, and serum creatinine levels in model 1 and UNOS MELD (2016) score, decompensation status, and serum albumin levels in model 2 (Table 3).

Next, we evaluated the prognostic performance of the previously provided cut-off of 1.4 in our outcome cohort. Importantly, this cut-off was not only associated with liver-related death in competing risk regression analysis (Table S2), but also hepatic decompensation (Cox regression Table 4 and Table S3; competing risk regression Table S4), as well as liver-related hospitalisation (Cox regression Table S5; competing risk regression Table S6), and ACLF in dACLD (Cox regression Table S7; competing risk regression Table S8).

In addition, we compared the prognostic performance of NH_3-ULN for hepatic decompensation/liver-related death and liver-related death to UNOS MELD (2016) score and HVPG in time-dependent AUROC analyses for the following time points: 12, 24, 36, 48, and 60 months. Regarding hepatic decompensation/liver-related death, HVPG showed significantly better discriminatory ability compared to NH_3-ULN at 24 months (NH_3-ULN *vs.* HVPG: *p* = 0.044 after accounting for multiplicity). In contrast, NH_3-ULN was comparable to time-dependent AUROC of the UNOS MELD (2016) score (Fig. 2A). Importantly, time-dependent AUROC of NH_3-ULN for liver-related death was comparable to those of UNOS MELD (2016)-score and HVPG at all tested time points (Fig. 2B).

Finally, stratifying the cohort according to NH_3-ULN quartiles (q1: <0.49, q2: 0.49–0.66, q3: 0.66–0.91, q4: ≥0.91) identified patient groups with a distinct prognosis (subdistribution HR [SHR] *p* <0.001; Fig. 3A and B). In line, the previously proposed cut-off identified individuals with particular poor outcomes in regard to liver-related death (SHR: 3.39 [95% CI: 2.08–5.53]; *p* <0.001; Fig. 3C) as well as hepatic decompensation/liver-related death (SHR: 4.11 [95% CI: 2.74–6.16]; *p* <0.001; Fig. 3D).

Associations between ammonia and disease-driving mechanisms in the biomarker cohort

Baseline characteristics of the biomarker cohort are provided in Table 1. Participants included in the biomarker cohort had less viral and more ARLD as underlying aetiology and were more often decompensated at baseline (62% *vs.* 46%; *p* <0.001).

Finally, we evaluated the correlation of ammonia with the severity of liver disease (UNOS MELD [2016] score and HVPG), serum BA levels, endothelial dysfunction (vWF), markers of systemic inflammation (CRP, PCT, and IL-6) as well as liver fibrogenesis/matrix remodelling (ELF-test), and markers of hyperdynamic circulation/systemic haemodynamic impairment (mean arterial pressure [MAP], copeptin, renin, and serum sodium).

As demonstrated in Fig. 4, ammonia showed low correlations with UNOS MELD (2016) score, BAs, HVPG, vWF, and ELF-test, as well as associations with CRP, IL-6, and PCT, MAP, renin, and serum sodium in the biomarker cohort. Further results on the correlations of ammonia with these biomarkers among compensated and decompensated individuals are reported in the Supplementary material.

Discussion

Although routinely measured ammonia is of limited value for diagnosing HE, a recent landmark study highlighted its prognostic implications. In our study, venous ammonia increased across clinical stages of ACLD as well as with more severe hepatic dysfunction and portal hypertension. Importantly, time-dependent AUROC values of ammonia for liver-related death were similar to the laboratory-based composite score UNOS MELD (2016) and HVPG, which can only be measured invasively. Ammonia was not only independently associated with liver-

Table 4. Uni- and multivariable Cox regression analyses of factors associated with hepatic decompensation/liver-related death including – among other parameters – CTP score, serum sodium, and creatinine (model 1) or serum UNOS MELD (2016) score, clinical stage, and serum albumin (model 2) in the outcome cohort.

Patient characteristics	Univariable		Model 1 (including CTP score, sodium, and creatinine)		Model 2 (including MELD and albumin)	
	HR (95% CI)	*p* value	aHR (95% CI)	*p* value	aHR (95% CI)	*p* value
Age, year	1.03 (1.02–1.05)	**<0.001**	1.02 (1.01–1.04)	**0.007**	1.02 (1.01–1.04)	**0.003**
HVPG, mmHg	1.15 (1.12–1.17)	**<0.001**	1.10 (1.07–1.13)	**<0.001**	1.09 (1.06–1.12)	**<0.001**
CTP score						
A	1		1		–	–
B	4.62 (3.33–6.43)	**<0.001**	2.22 (1.52–3.25)	**<0.001**	–	–
C	6.91 (4.33–11.04)	**<0.001**	2.47 (1.38–4.40)	**0.002**	–	–
UNOS MELD (2016) score, point	1.12 (1.09–1.15)	**<0.001**	–	–	0.99 (0.96–1.03)	0.713
Decompensated *vs.* compensated ACLD	5.63 (3.96–8.00)	**<0.001**	–	–	2.35 (1.58–3.49)	**<0.001**
Sodium, mmol/L	0.90 (0.87–0.93)	**<0.001**	0.98 (0.94–1.03)	0.443	–	–
Creatinine, mg/dl	2.49 (1.52–4.06)	**<0.001**	1.38 (0.84–2.26)	0.199	–	–
Albumin, g/L	0.89 (0.86–0.91)	**<0.001**	–	–	0.95 (0.92–0.98)	**<0.001**
CRP, mg/L	2.47 (1.98–3.08)	**<0.001**	1.69 (1.28–2.23)	**<0.001**	1.64 (1.24–2.16)	**<0.001**
NH_3-ULN ≥1.4 *vs.* <1.4	3.84 (2.58–5.71)	**<0.001**	2.08 (1.35–3.22)	**<0.001**	1.99 (1.31–3.02)	**0.001**
Concordance ± SE				**0.819 ± 0.015**		**0.825 ± 0.014**
AIC				**1,819.569**		**1,807.099**

Values of *p* in bold denote *p* <0.05.
ACLD, advanced chronic liver disease; aHR, adjusted hazard ratio; AIC, Akaike information criterion; ARLD, alcohol-related liver disease; CRP, C-reactive protein; CTP, Child–Turcotte–Pugh score; HVPG, hepatic venous pressure gradient; NAFLD, non-alcoholic fatty liver disease; NH_3-ULN, ammonia adjusted for the upper limit of normal; UNOS MELD (2016) score, United Network for Organ Sharing Model of end-stage liver disease (2016) score.

related death – as shown previously – but also with other liver-related outcomes including ACLF, even after adjusting for liver disease, systemic inflammation, and portal hypertension severity. Notably, our findings support the use of the previously proposed cut-off of NH_3-ULN of ≥1.4 for risk stratification. Finally, we have provided information on associated pathophysiologic mechanisms that may explain the prognostic value of ammonia, that is, liver fibrogenesis/matrix remodelling and endothelial dysfunction.

Interestingly, diabetes was independently associated with venous plasma ammonia levels, even after adjusting for various co-factors (adjusted B: 0.134 [95% CI: 0.016, 0.252]; $p = 0.026$; Table 2). Diabetes may increase venous plasma ammonia levels by autonomic dysfunction, extended gastrointestinal transit times, and bacterial overgrowth as well as increased protein catabolism and accelerated muscle-breakdown.[23] Even in earlier stages of fibrosis in individuals with NAFLD/metabolic-associated fatty liver disease (MAFLD), deficiencies in urea synthesis (in part caused by impaired liver-α-cell axis with glucagon resistance and impaired ureagenesis[24]), microglial activation, astrocyte swelling, and possibly even neurodegenerative changes and brain atrophy – all caused by elevated ammonia levels – have been reported.[25,26]

From a clinical perspective, Haj and Rockey[27] have found that in individuals with cirrhosis hospitalised with HE, ammonia was often normal and did not impact treatment decisions, thereby arguing against the routine use of ammonia as either an initial diagnostic test or for guiding medical therapy.

However, moving to risk stratification, ammonia is increasingly acknowledged as a prognostic biomarker. Vierling *et al.*[28] have shown that ammonia was able to identify people at risk for HE-related events. Moreover, a recent multicentre study by Tranah *et al.*[15] indicated that ammonia is predictive of hospitalisation/liver-related complications and mortality, showing a better prognostic performance than traditional scores. To corroborate the main finding of this study, we have externally validated the cut-off of ≥1.4 NH_3-ULN. However, the authors did neither adjust their multivariable models for systemic inflammation nor portal hypertension severity, which has been accounted for in our work. Notably, ammonia levels varied significantly throughout the study centres, with only one centre reporting ammonia levels that were comparable to our cohort, which may be explained by differences in patient selection and characteristics. Interestingly, ammonia levels reported by Gairing *et al.*[29] were similar to ours and only a minority of individuals had ammonia levels above the ULN (13% *vs.* 19% in our cohort).

In people admitted because of acute decompensation of cirrhosis, ammonia levels have been found to be independently associated with mortality.[30] Our study extends these findings, as it demonstrated that stratifying individuals according to the proposed cut-off of ≥1.4 NH_3-ULN identifies decompensated individuals at risk for ACLF, even if they are still outpatients/clinically stable. Therefore, it may provide the opportunity for the timely initiation of disease-modifying interventions that are currently under investigation (e.g. LIVERHOPE, NCT03150459).

Hyperammonaemia in ACLD is driven by ammonia overproduction/altered microbiome in the intestinal tract,[6,7] decreased metabolic capacity in the hepatocytes leading to a reduced ammonia metabolism in the urea cycle and via glutamine synthetase,[8,9] and portal hypertension through the development of portosystemic shunting via collateral flow.[10,11] Effects of hyperammonaemia include immune dysfunction and

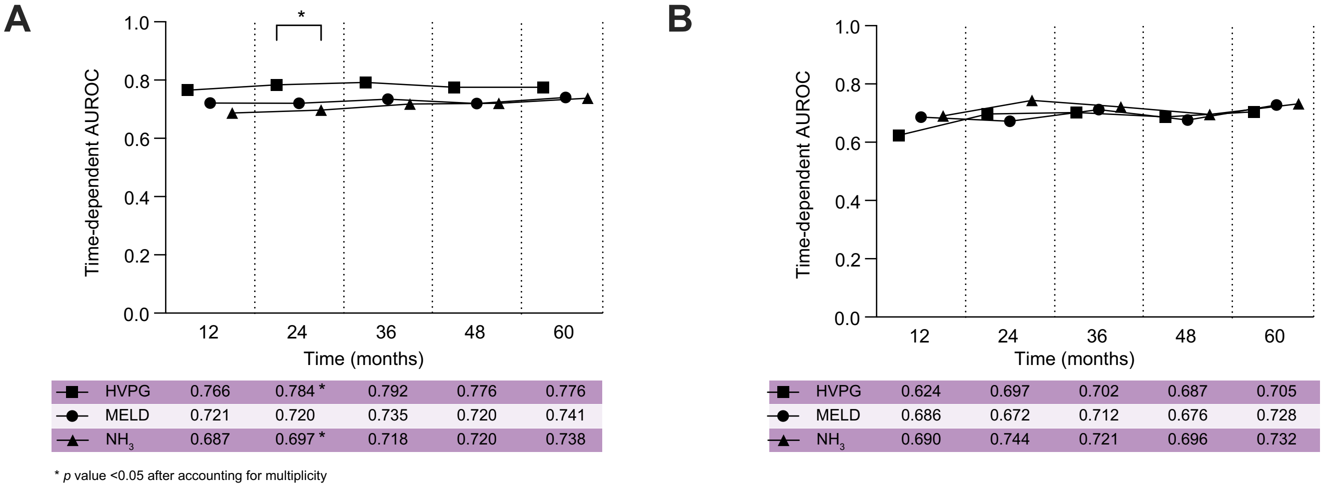

Fig. 2. Prognostic implications of NH_3-ULN compared to HVPG and UNOS MELD (2016). Univariable time-dependent area under the receiver operating curve (AUROC) analyses comparing the prognostic performances of UNOS MELD (2016), HVPG, and NH_3-ULN for prognostication of (A) hepatic decompensation/liver-related death and (B) liver-related death in the outcome cohort. HVPG, hepatic venous pressure gradient; NH_3-ULN, ammonia adjusted for the upper limit of normal; UNOS MELD (2016) score, United Network for Organ Sharing Model of end-stage liver disease (2016) score.

sarcopenia as well as direct negative implications on liver disease progression.[10] Recent works on the impact of hyperammonaemia in animal and *in-vitro* studies on fibrosis demonstrated the induction of oxidative stress and apoptosis and the activation of hepatic stellate cells (HSCs).[31,32] Intriguingly, we observed consistent (*i.e.* both in cACLD and dACLD) positive correlations between ammonia and the ELF-test, which has been shown to reflect fibrogenesis/extracellular matrix remodelling irrespective of the stage of ACLD and indicate HSC activation.[33] Moreover, there were also positive correlations with systemic inflammation, vWF as a marker of endothelial dysfunction, and severity of hepatic dysfunction and portal hypertension. Finally, ammonia

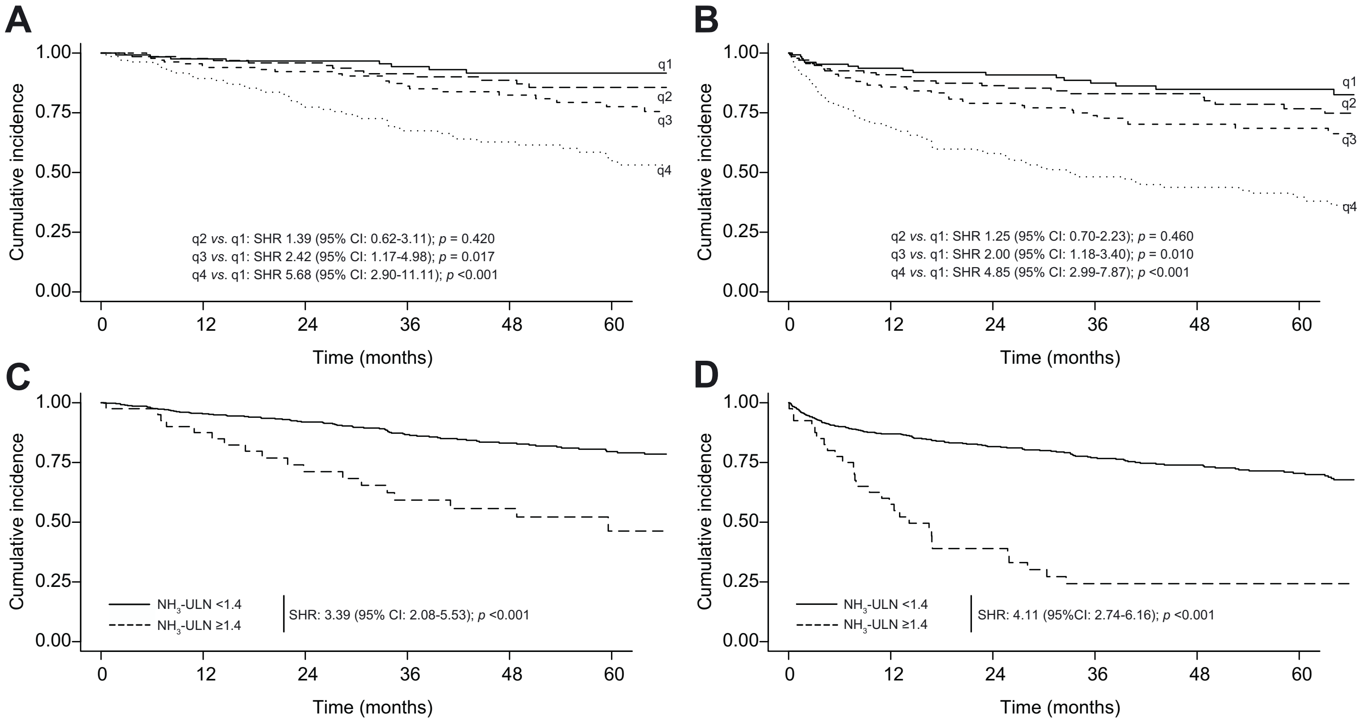

Fig. 3. Stratification of outcomes according to NH_3-ULN. Cumulative incidence plots of remaining free of liver-related death (A,C) and hepatic decompensation/liver-related death (B,D) according to NH_3-ULN quartiles (A,B) and previously published cut-off of 1.4 (C,D) in the outcome cohort. For these cumulative incidence plots, competing risk curves were depicted. In A and B, the subdistribution hazard ratios (SHR) were calculated. For C and D, individuals with <1.4 *vs.* ≥1.4 NH_3-ULN were compared. NH_3-ULN, ammonia adjusted for the upper limit of normal; q, quartile.

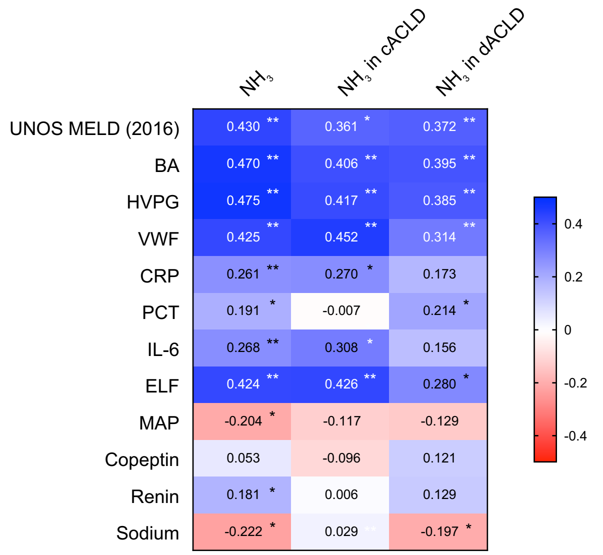

Fig. 4. Heatmaps of correlations of NH_3 and pathophysiological biomarkers in the biomarker cohort. **p* <0.05; ***p* <0.001; Spearman's correlation analyses were conducted. ACLD, advanced chronic liver disease; BA, bile acid; CRP, C-reactive protein; ELF, enhanced liver fibrosis; HVPG, hepatic venous pressure gradient; IL-6, interleukin-6; MAP, mean arterial pressure; NH_3, ammonia; PCT, procalcitonin; UNOS MELD (2016), United Network for Organ Sharing model for end-stage liver disease (2016); vWF, von Willebrand factor antigen.

also correlated with BAs, which may be interpreted as a biomarker for portosystemic shunting,[34] which has also been linked with disease progression.[35,36]

The main limitation of our study is its retrospective design. However, participants were thoroughly characterised at the time of HVPG measurement. For our multivariable models, we were unable to consider several potentially important prognostic indicators (*e.g.* sarcopaenia/frailty), as they have not been recorded systematically. Nevertheless, participants included in our study were extensively characterised in terms of portal hypertension severity, prognostic scores, and routine laboratory parameters including markers of systemic inflammation – importantly, all of these aspects have been considered in our analyses. Model selection was based on expert opinion/biological relevance. Applying backward elimination for variable selection yielded a 'slimmer' model for predictive purposes, that still included NH_3. Furthermore, we cannot exclude that some hepatic decompensation events have been missed. However, we have thoroughly reviewed electronic health records of the Vienna hospital association and nationwide electronic health records. Moreover, we have also performed searches of the liver transplant database of our institution (*i.e.* the only transplant centre in eastern Austria) and examined the nationwide death registry. As complete information on (reason of) death is guaranteed by the latter measure, we included liver-related death in all composite endpoints to ensure the ascertainment of the most severe disease courses. We cannot rule out selection bias because we only included people undergoing HVPG measurement. However, haemodynamic evaluations are routinely performed for risk stratification and treatment monitoring purposes at our centre, and thus, we are confident that our study population is quite representative of clinically stable outpatients with ACLD treated at our centre. Finally, ammonia testing has several limitations. There is substantial laboratory variability[37] and arterial ammonia might be preferred over venous ammonia.[38–40] However, it has been shown that venous ammonia closely correlates with arterial ammonia in people with cirrhosis[41] and venous sampling substantially increases feasibility and therefore clinical utility in outpatients. We have provided a detailed description of the measurement of ammonia in the Materials and methods section of this study and are confident that our results are reliable, because preanalytical and analytical conditions were highly standardised.

In conclusion, venous ammonia predicts hepatic decompensation, non-elective liver-related hospitalisation, ACLF, and liver-related death, independently of established prognostic indicators including CRP and HVPG.

Although venous ammonia is linked with several key disease-driving mechanisms, its prognostic value is not explained by associated hepatic dysfunction, systemic inflammation, or portal hypertension severity, suggesting direct toxicity.

Abbreviations

ACLD, advanced chronic liver disease; ACLF, acute-on-chronic liver failure; aHR, adjusted hazard ratio; ARLD, alcohol-related liver disease; AUROC, area under the receiver operating characteristic curve; BAs, Bile acids; CRP, C-reactive protein; CTP, Child–Turcotte–Pugh score; ELF®-test, enhanced liver fibrosis-test; HE, hepatic encephalopathy; HSC, hepatic stellate cell; HVPG, hepatic venous pressure gradient; MAFLD, metabolic-associated fatty liver disease; MAP, mean arterial pressure; NAFLD, non-alcoholic fatty liver disease; NH_3-ULN, ammonia-adjusted for the upper limit of normal; PCT, procalcitonin; SHR, subdistribution hazard ratio; UNOS MELD (2016), United Network for Organ Sharing model for end-stage liver disease (2016); vWF, von Willebrand factor.

Financial support

The authors received no financial support to produce this manuscript.

Conflicts of interest

The authors have nothing to disclose regarding the work under consideration for publication. LB, JK, GS, MJ, LH, AFS, PS, and TS have nothing to disclose. The following authors disclose conflicts of interests outside the submitted work. RP received travel support from AbbVie, Gilead and Takeda. BSc received travel support from AbbVie, Ipsen and Gilead. BSi received travel support from AbbVie and Gilead. MP served as a speaker and/or consultant and/or advisory board member for Bayer, Bristol-Myers Squibb, Eisai, Ipsen, Lilly, MSD, and Roche, and received travel support from Bayer and Bristol-Myers Squibb. MT served as a speaker and/or consultant and/or advisory board member for Albireo, BiomX, Falk, Boehringer Ingelheim, Bristol-Myers Squibb, Falk, Genfit, Gilead, Hightide, Intercept, Janssen, MSD, Novartis, Phenex, Pliant, Regulus, Siemens and Shire, and received travel support from AbbVie, Falk, Gilead, and Intercept as well as grants/research support from Albireo, Alnylam, Cymabay, Falk, Gilead, Intercept, MSD, Takeda, and UltraGenyx. He is also co-inventor of patents on the medical use of 24-norursodeoxycholic acid. TR received grant support from AbbVie, Boehringer-Ingelheim, Gilead, Intercept, MSD, Myr Pharmaceuticals, Philips Healthcare, Pliant, Siemens, and W. L. Gore & Associates; speaking honoraria from AbbVie, Gilead, Gore, Intercept, Roche, and MSD; consulting/advisory board fees from AbbVie, Bayer, Boehringer-Ingelheim, Gilead, Intercept, MSD, and Siemens; and travel support from AbbVie, Boehringer-Ingelheim, Gilead, and Roche. MM served as a speaker and/or consultant and/or advisory board member for AbbVie, Collective Acumen, Gilead, Takeda, and W. L. Gore & Associates and received travel support from AbbVie and Gilead.

Please refer to the accompanying ICMJE disclosure forms for further details.

Authors' contributions

Concept of the study: LB, JK, MM. Data collection: LB, JK, RP, BSi, BSc, MM. Statistical analysis: LB, JK, MM. Drafting of the manuscript: LB, JK, MM. Revision for important intellectual content and approval of the final manuscript: all authors.

Data availability statement

The data that support the findings of this study are available from the corresponding author upon reasonable request.

Supplementary data

Supplementary data to this article can be found online at https://doi.org/10.1016/j.jhepr.2023.100682.

References

Author names in bold designate shared co-first authorship

[1] Eslam M, Sanyal AJ, George J. MAFLD: a consensus-driven proposed nomenclature for metabolic associated fatty liver disease. Gastroenterology 2020;158:1999–2014.e1.

[2] Sanyal AJ, Van Natta ML, Clark J, Neuschwander-Tetri BA, Diehl A, Dasarathy S, et al. Prospective study of outcomes in adults with nonalcoholic fatty liver disease. N Engl J Med 2021;385:1559–1569.

[3] Mandorfer M, Simbrunner B. Prevention of first decompensation in advanced chronic liver disease. Clin Liver Dis 2021;25:291–310.

[4] Trebicka J, Fernandez J, Papp M, Caraceni P, Laleman W, Gambino C, et al. The PREDICT study uncovers three clinical courses of acutely decompensated cirrhosis that have distinct pathophysiology. J Hepatol 2020;73:842–854.

[5] **Costa D**, **Simbrunner B**, Jachs M, Hartl L, Bauer D, Paternostro R, et al. Systemic inflammation increases across distinct stages of advanced chronic liver disease and correlates with decompensation and mortality. J Hepatol 2021;74:819–828.

[6] Rai R, Saraswat VA, Dhiman RK. Gut microbiota: its role in hepatic encephalopathy. J Clin Exp Hepatol 2015;5(Suppl 1):S29–S36.

[7] Romero-Gómez M, Ramos-Guerrero R, Grande L, de Terán LC, Corpas R, Camacho I, et al. Intestinal glutaminase activity is increased in liver cirrhosis and correlates with minimal hepatic encephalopathy. J Hepatol 2004;41:49–54.

[8] Fleming KE, Wanless IR. Glutamine synthetase expression in activated hepatocyte progenitor cells and loss of hepatocellular expression in congestion and cirrhosis. Liver Int 2013;33:525–534.

[9] Wierling C. Bridging the gap between metabolic liver processes and functional tissue structure by integrated spatiotemporal modeling applied to hepatic ammonia detoxification. Hepatology 2014;60:1823–1825.

[10] Deutsch-Link S, Moon AM. The ongoing debate of serum ammonia levels in cirrhosis: the good, the bad, and the ugly. Am J Gastroenterol 2023;118:10–13.

[11] Tapper EB, Jiang ZG, Patwardhan VR. Refining the ammonia hypothesis: a physiology-driven approach to the treatment of hepatic encephalopathy. Mayo Clin Proc 2015;90:646–658.

[12] EASL Clinical Practice Guidelines on the management of hepatic encephalopathy. J Hepatol 2022;77:807–824.

[13] Angelova PR, Kerbert AJC, Habtesion A, Hall A, Abramov AY, Jalan R. Hyperammonaemia induces mitochondrial dysfunction and neuronal cell death. JHEP Rep 2022;4:100510.

[14] Hadjihambi A, Cudalbu C, Pierzchala K, Simicic D, Donnelly C, Konstantinou C, et al. Abnormal brain oxygen homeostasis in an animal model of liver disease. JHEP Rep 2022;4:100509.

[15] **Tranah TH**, **Ballester MP**, **Carbonell-Asins JA**, Ampuero J, Alexandrino G, Caracostea A, et al. Plasma ammonia levels predict hospitalisation with liver-related complications and mortality in clinically stable outpatients with cirrhosis. J Hepatol 2022;77:1554–1563.

[16] Ferlitsch A, Bota S, Paternostro R, Reiberger T, Mandorfer M, Heinisch B, et al. Evaluation of a new balloon occlusion catheter specifically designed for measurement of hepatic venous pressure gradient. Liver Int 2015;35:2115–2120.

[17] Reiberger T, Schwabl P, Trauner M, Peck-Radosavljevic M, Mandorfer M. Measurement of the hepatic venous pressure gradient and transjugular liver biopsy. J Vis Exp 2020;160:e58819.

[18] de Franchis R, Bosch J, Garcia-Tsao G, Reiberger T, Ripoll C. Baveno VII – renewing consensus in portal hypertension. J Hepatol 2022;76:959–974.

[19] D'Amico G, Morabito A, D'Amico M, Pasta L, Malizia G, Rebora P, et al. Clinical states of cirrhosis and competing risks. J Hepatol 2018;68:563–576.

[20] Moreau R, Jalan R, Gines P, Pavesi M, Angeli P, Cordoba J, et al. Acute-on-chronic liver failure is a distinct syndrome that develops in patients with acute decompensation of cirrhosis. Gastroenterology 2013;144:1426–1437.

[21] Fine JP, Gray RJ. A proportional hazards model for the subdistribution of a competing risk. J Am Stat Assoc 1999;94:496–509.

[22] Therneau T, Crowson C, Atkinson E. Using time dependent covariates and time dependent coefficients in the Cox Model. 2022; https://cran.r-project.org/web/packages/survival/vignettes/timedep.pdf. [Accessed 24 January 2023].

[23] Gairing SJ, Schleicher EM, Labenz C. Diabetes mellitus – risk factor and potential future target for hepatic encephalopathy in patients with liver cirrhosis? Metab Brain Dis 2022. https://doi.org/10.1007/s11011-022-01068-4.

[24] Richter MM, Galsgaard KD, Elmelund E, Knop FK, Suppli MP, Holst JJ, et al. The liver-α-cell axis in health and in disease. Diabetes 2022;71:1852–1861.

[25] Kjærgaard K, Mikkelsen ACD, Wernberg CW, Grønkjær LL, Eriksen PL, Damholdt MF, et al. Cognitive dysfunction in non-alcoholic fatty liver disease-current knowledge, mechanisms and perspectives. J Clin Med 2021;10:673.

[26] Weinstein G, Zelber-Sagi S, Preis SR, Beiser AS, DeCarli C, Speliotes EK, et al. Association of nonalcoholic fatty liver disease with lower brain volume in healthy middle-aged adults in the Framingham study. JAMA Neurol 2018;75:97–104.

[27] Haj M, Rockey DC. Ammonia Levels do not guide clinical management of patients with hepatic encephalopathy caused by cirrhosis. Am J Gastroenterol 2020;115:723–728.

[28] Vierling JM, Mokhtarani M, Brown Jr RS, Mantry P, Rockey DC, Ghabril M, et al. Fasting blood ammonia predicts risk and frequency of hepatic encephalopathy episodes in patients with cirrhosis. Clin Gastroenterol Hepatol 2016;14:903–906.e1.

[29] Gairing SJ, Kaps L, Schleicher EM, Galle PR, Labenz C. Ammonia – an old friend with a new area of application. J Hepatol 2023;78:e22–e23.

[30] Shalimar, Sheikh MF, Mookerjee RP, Agarwal B, Acharya SK, Jalan R. Prognostic role of ammonia in patients with cirrhosis. Hepatology 2019;70:982–994.

[31] De Chiara F, Thomsen KL, Habtesion A, Jones H, Davies N, Gracia-Sancho J, et al. Ammonia scavenging prevents progression of fibrosis in experimental nonalcoholic fatty liver disease. Hepatology 2020;71:874–892.

[32] Jia B, Yu ZJ, Duan ZF, Lü XQ, Li JJ, Liu XR, et al. Hyperammonaemia induces hepatic injury with alteration of gene expression profiles. Liver Int 2014;34:748–758.

[33] Simbrunner B, Villesen IF, Königshofer P, Scheiner B, Bauer D, Paternostro R, et al. Systemic inflammation is linked to liver fibrogenesis in patients with advanced chronic liver disease. Liver Int 2022;42:2501–2512.

[34] Simbrunner B, Trauner M, Reiberger T. Review article: therapeutic aspects of bile acid signalling in the gut-liver axis. Aliment Pharmacol Ther 2021;54:1243–1262.

[35] **Praktiknjo M**, **Simón-Talero M**, Römer J, Roccarina D, Martínez J, Lampichler K, et al. Total area of spontaneous portosystemic shunts independently predicts hepatic encephalopathy and mortality in liver cirrhosis. J Hepatol 2020;72:1140–1150.

[36] Simón-Talero M, Roccarina D, Martínez J, Lampichler K, Baiges A, Low G, et al. Association between portosystemic shunts and increased complications and mortality in patients with cirrhosis. Gastroenterology 2018;154:1694–1705.e4.

[37] Dam G, Ott P, Aagaard NK, Vilstrup H. Branched-chain amino acids and muscle ammonia detoxification in cirrhosis. Metab Brain Dis 2013;28:217–220.

[38] Brusilow SW, Koehler RC, Traystman RJ, Cooper AJ. Astrocyte glutamine synthetase: importance in hyperammonemic syndromes and potential target for therapy. Neurotherapeutics 2010;7:452–470.

[39] da Fonseca-Wollheim F. Preanalytical increase of ammonia in blood specimens from healthy subjects. Clin Chem 1990;36:1483–1487.

[40] Kramer L, Tribl B, Gendo A, Zauner C, Schneider B, Ferenci P, et al. Partial pressure of ammonia versus ammonia in hepatic encephalopathy. Hepatology 2000;31:30–34.

[41] Ong JP, Aggarwal A, Krieger D, Easley KA, Karafa MT, Van Lente F, et al. Correlation between ammonia levels and the severity of hepatic encephalopathy. Am J Med 2003;114:188–193.

Journal of Hepatology, Volume ■

Supplemental information

Impact of ammonia levels on outcome in clinically stable outpatients with advanced chronic liver disease

Lorenz Balcar, Julia Krawanja, Bernhard Scheiner, Rafael Paternostro, Benedikt Simbrunner, Georg Semmler, Mathias Jachs, Lukas Hartl, Albert Friedrich Stättermayer, Philipp Schwabl, Matthias Pinter, Thomas Szekeres, Michael Trauner, Thomas Reiberger, and Mattias Mandorfer

Impact of ammonia levels on outcome in clinically stable outpatients with advanced chronic liver disease

Lorenz Balcar, Julia Krawanja, Bernhard Scheiner, Rafael Paternostro, Benedikt Simbrunner, Georg Semmler, Mathias Jachs, Lukas Hartl, Albert Friedrich Stättermayer, Philipp Schwabl, Matthias Pinter, Thomas Szekeres, Michael Trauner, Thomas Reiberger, Mattias Mandorfer

Table of contents

Supplementary results 2

Fig. S1 3

Fig. S2 5

Table S1 6

Table S2 8

Table S3 10

Table S4 .... **Error! Bookmark not defined.**

Table S5 .... **Error! Bookmark not defined.**

Table S6 .... **Error! Bookmark not defined.**

Table S7 .... **Error! Bookmark not defined.**

Table S8 20

Supplementary results

Associations between ammonia and biomarkers in compensated/decompensated ACLD patients in the pathophysiology cohort

As demonstrated in Figure 5, ammonia showed low correlations with UNOS MELD (2016)-score (Spearman's ρ=0.361; p=0.002), HVPG (ρ=0.417; p<0.001), VWF (ρ=0.452; p<0.001), ELF-test (ρ=0.426; p<0.001), IL-6 (ρ=0.308; p=0.008), and BA (ρ=0.406; p<0.001), as well as associations with CRP (ρ=0.270; p=0.020), and LBP (ρ=-0.268; p=0.021) in patients with compensated ACLD.

In patients with decompensated ACLD, ammonia showed low correlation with UNOS MELD (2016)-score (Spearman's ρ=0.372; p<0.001), HVPG (ρ=0.385; p<0.001), VWF (ρ=0.314; p<0.001), and BA (ρ=0.395; p<0.001), as well as associations with PCT (ρ=0.214; p=0.020), ELF-test (ρ=0.280; p=0.002) and serum sodium (ρ=-0.197; p=0.031).

Supplementary figures

Fig. S1

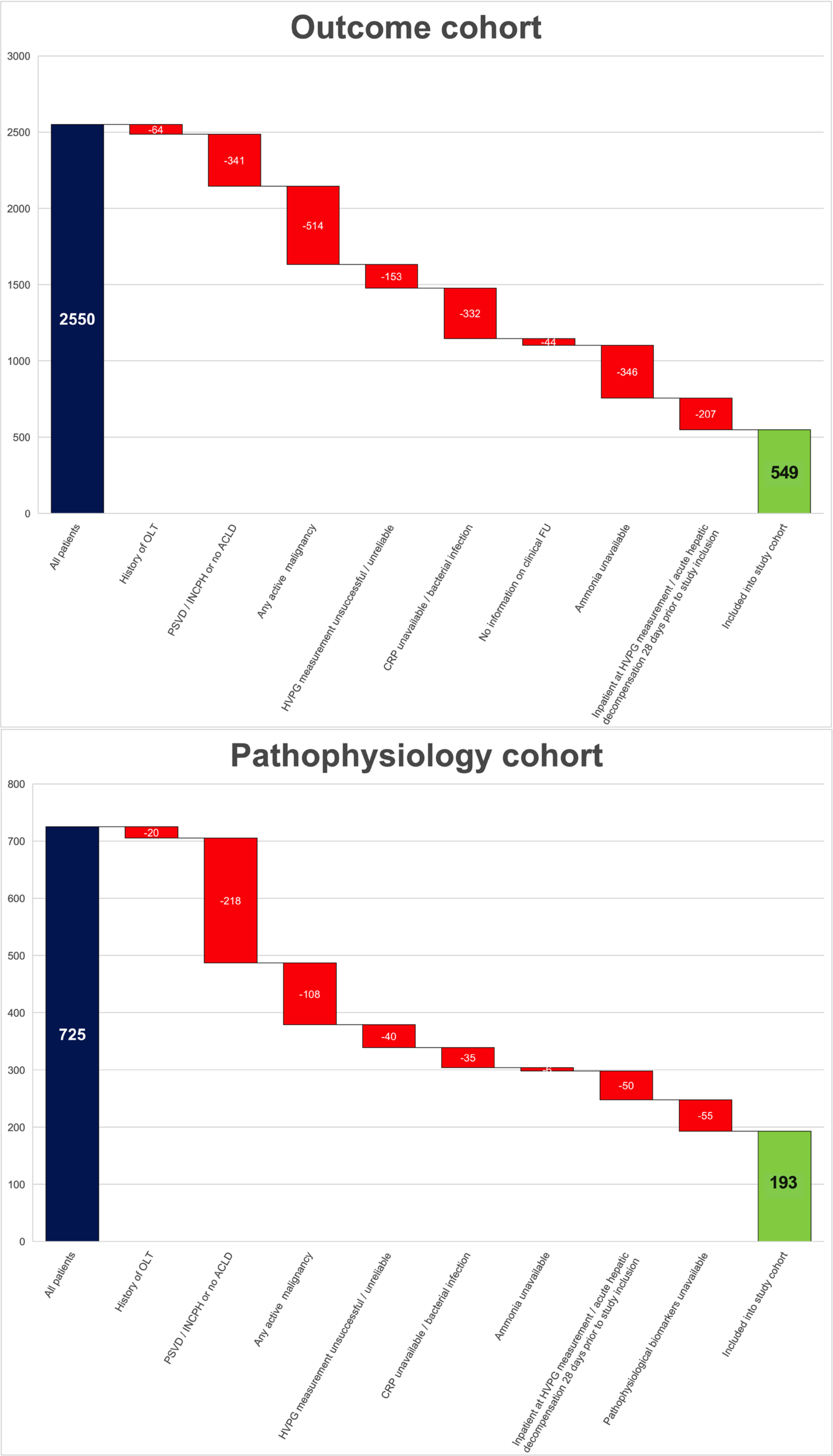

Fig. S1. Patient flow-chart showing the application of in- and exclusion.

Abbreviations: ACLD advanced chronic liver disease; CRP C-reactive protein; FU follow-up; HVPG hepatic venous pressure gradient; INCPH idiopathic non-cirrhotic portal hypertension; PSVD porto-sinusoidal vascular disorder; OLT orthotopic liver transplantation

Fig. S2

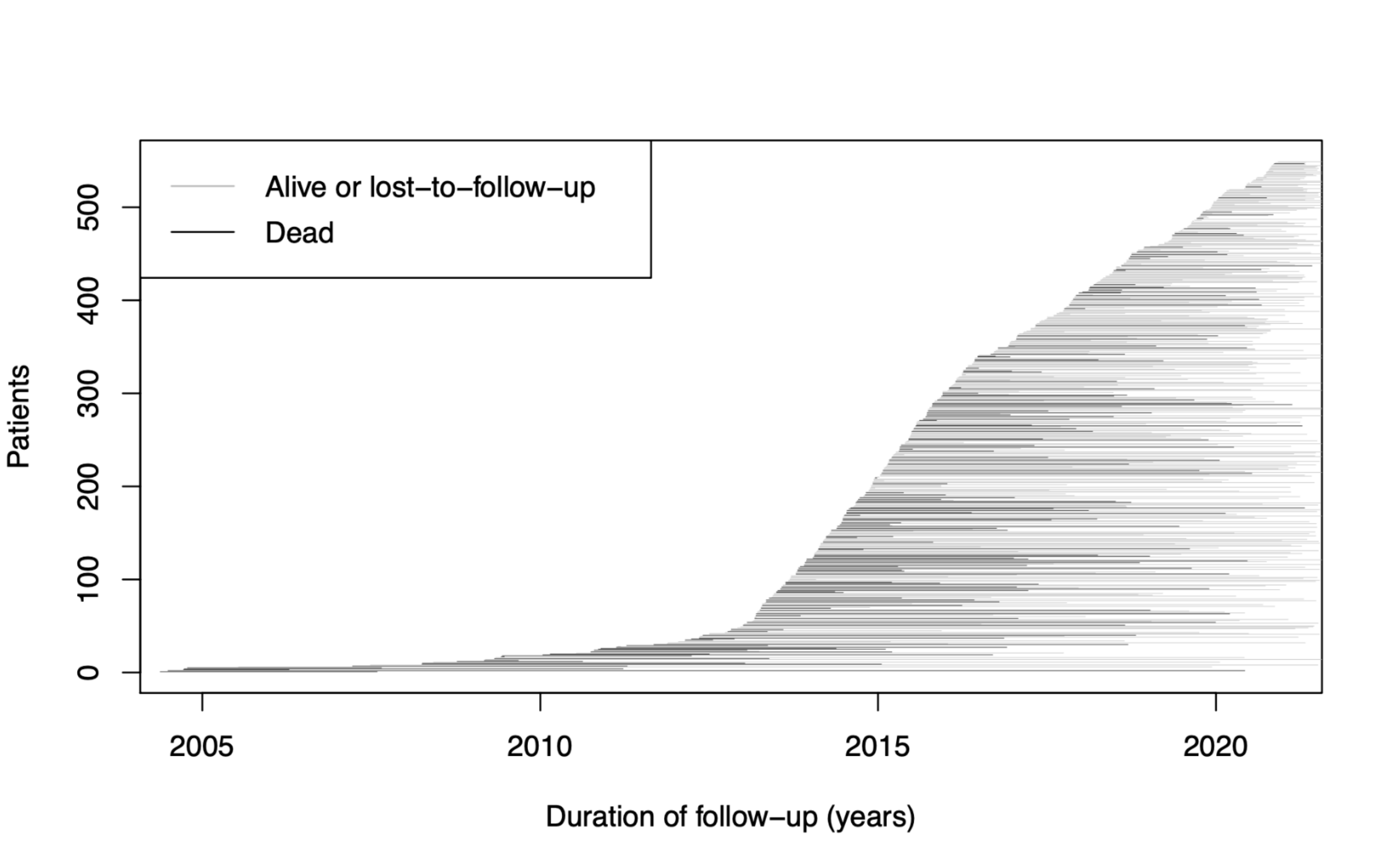

Fig. S2. Recruitment and follow-up over the study period.

Supplementary tables

Table S1

Disease severity indices	**Number of patients, n (%)**	**NH3, μmol x L^{-1}, median (IQR)**	**p-value**
CTP score			
A	342 (62%)	33.8 (25.8-45.3)	**<0.001**
B	168 (31%)	44.9 (35.8-58.8)	
C	39 (7%)	52.3 (37.4-80.3)	
UNOS MELD (2016) score			
<10	208 (38%)	31.3 (23.4-40.0)	**<0.001**
10-14	216 (39%)	41.5 (30.5-54.8)	
≥15	125 (23%)	46.4 (36.5-64.5)	
HVPG, mmHg			
HVPG 0-5 mmHg	42 (8%)	28.3 (24.0-37.1)	**<0.001**
HVPG 6-9 mmHg	83 (15%)	30.7 (22.0-35.8)	
HVPG 10-15 mmHg	137 (25%)	35.5 (26.8-46.0)	
HVPG ≥16 mmHg	287 (52%)	44.8 (33.8-59.6)	
Clinical Stages			
Probable cACLD	42 (8%)	28.3 (24.0-37.1)	**<0.001**
CS 0	74 (14%)	29.8 (21.7-35.2)	
CS 1	185 (34%)	35.8 (27.6-47.1)	
CS 2	25 (5%)	45.3 (33.7-60.0)	
CS 3	132 (24%)	40.1 (30.1-56.9)	
CS 4	91 (17%)	49.9 (40.3-68.7)	
Substages of ACLD			
Compensated	297 (54%)	33.6 (25.6-43.1)	-
SDC	209 (38%)	45.8 (34.7-62.0)	0.934
UDC	36 (7%)	46.4 (30.7-58.3)	
Pre-ACLF	7 (1%)	48.7 (36.1-54.7)	

Table S1. Comparison of NH3 levels according to different liver disease severity indicators.

Abbreviations: AMM-ULN ammonia level corrected to the upper limit of normal; *cACLD compensated advanced chronic liver disease; CS clinical stage; CTP Child-Turcotte-Pugh; dACLD decompensated advanced chronic liver disease; HVPG hepatic venous pressure gradient; UNOS MELD (2016) United Network for Organ Sharing model for end-stage liver disease (2016)*

Table S2

Patient characteristics	**Univariable**		**Model 1 (incl. CTP score, sodium, and creatinine)**		**Model 2 (incl. MELD, and albumin)**	
	**SHR (95%CI)**	**p-value**	**aSHR (95%CI)**	**p-value**	**aSHR (95%CI)**	**p-value**
Age, year	1.05 (1.03-1.08)	**<0.001**	1.06 (1.03-1.08)	**<0.001**	1.05 (1.03-1.08)	**<0.001**
HVPG, mmHg	1.07 (1.05-1.10)	**<0.001**	1.03 (0.99-1.07)	0.110	1.03 (0.99-1.07)	0.120
CTP score						
A	1		1		-	-
B	2.69 (1.75-4.13)	**<0.001**	1.64 (0.94-2.86)	0.085	-	-
C	4.67 (2.70-8.10)	**<0.001**	2.35 (1.04-5.33)	**0.040**	-	-
UNOS MELD (2016) score, point	1.09 (1.05-1.13)	**<0.001**	-	-	1.03 (0.98-1.08)	0.260
Decompensated vs. compensated ACLD	2.39 (1.59-3.58)	**<0.001**	-	-	0.98 (0.56-1.69)	0.940
Sodium, mmol x L^{-1}	0.93 (0.88-0.98)	**0.004**	0.99 (0.93-1.06)	0.850	-	-
Creatinine, mg x dL^{-1}	2.07 (1.16-3.70)	**0.014**	0.91 (0.45-1.82)	0.790	-	-
Albumin, g x L^{-1}	0.92 (0.90-0.95)	**<0.001**	-	-	0.97 (0.94-1.00)	0.078
CRP, mg x L^{-1}	2.02 (1.54-2.63)	**<0.001**	1.50 (1.09-2.06)	**0.014**	1.48 (1.05-2.07)	**0.024**
NH3-ULN ≥1.4 vs. <1.4	3.39 (2.08-5.53)	**<0.001**	2.19 (1.22-3.94)	**0.009**	2.41 (1.32-4.40)	**0.004**

Table S2. Competing risk regression analyses of factors associated with liver-related death including – among other parameters – CTP score, serum sodium, and creatinine **(model 1)** or UNOS MELD (2016) score, clinical stage, and serum albumin **(model 2)** with requirement of liver transplantation/non-liver-related death/removal of the primary etiological factor as competing risks in the **outcome cohort**.

Abbreviations: ACLD advanced chronic liver disease; aHR adjusted hazard ratio; NH3-ULN ammonia adjusted for the upper limit of normal; CI confidence interval; CRP C-reactive protein; CTP Child-Turcotte-Pugh score; HVPG hepatic venous pressure gradient; UNOS MELD (2016) United Network for Organ Sharing Model of End-stage Liver Disease (2016)

Table S3

Patient characteristics	Univariable		Model 1 (incl. CTP score, sodium, and creatinine)		Model 2 (incl. MELD, and albumin)	
	**HR (95%CI)**	**p-value**	**aHR (95%CI)**	**p-value**	**aHR (95%CI)**	**p-value**
Age, year	1.02 (1.01-1.03)	**0.008**	1.01 (0.99-1.02)	0.349	1.01 (0.99-1.02)	0.282
HVPG, mmHg	1.13 (1.11-1.16)	**<0.001**	1.10 (1.07-1.14)	**<0.001**	1.10 (1.07-1.14)	**<0.001**
CTP score						
A	1		1		-	-
B	3.92 (2.82-5.45)	**<0.001**	1.92 (1.30-2.82)	**<0.001**	-	-
C	4.56 (2.70-7.70)	**<0.001**	1.39 (0.73-2.66)	0.315	-	-
UNOS MELD (2016) score, point	1.10 (1.07-1.13)	**<0.001**	-	-	0.99 (0.95-1.03)	0.540
Decompensated vs. compensated ACLD	3.42 (2.46-4.75)	**<0.001**	-	-	1.47 (1.01-2.13)	**0.043**
Sodium, mmol x L^{-1}	0.91 (0.88-0.95)	**<0.001**	1.00 (0.95-1.04)	0.908	-	-
Creatinine, mg x dL^{-1}	1.73 (1.01-2.99)	**0.048**	1.18 (0.69-2.01)	0.550	-	-
Albumin, g x L^{-1}	0.90 (0.88-0.93)	**<0.001**	-	-	0.96 (0.93-0.99)	**0.020**
CRP, mg x L^{-1}	2.23 (1.76-2.81)	**<0.001**	1.63 (1.22-2.19)	**0.001**	1.61 (1.21-2.16)	**0.001**
NH3-ULN ≥1.4 vs. <1.4	3.57 (2.35-5.41)	**<0.001**	2.38 (1.51-3.73)	**<0.001**	2.19 (1.41-3.40)	**<0.001**
**Concordance ± SE**			**0.779 ± 0.019**		**0.775 ± 0.019**	
**AIC**			**1749.919**		**1751.330**	

Table S3. Uni- and multivariable Cox regression analyses of factors associated with hepatic decompensation including – among other parameters – CTP score, serum sodium, and creatinine (model 1) or UNOS MELD (2016) score, clinical stage, and serum albumin (model 2).

Abbreviations: ACLD advanced chronic liver disease; aHR adjusted hazard ratio; AIC Akaike information criterion; NH3-ULN ammonia adjusted for the upper limit of normal; CI confidence interval; CRP C-reactive protein; CTP Child-Turcotte-Pugh score; HVPG hepatic venous pressure gradient; UNOS MELD (2016) United Network for Organ Sharing Model of End-stage Liver Disease (2016)

Table S4

Patient characteristics	**Univariable**		**Model 1 (incl. CTP score, sodium, and creatinine)**		**Model 2 (incl. MELD, and albumin)**	
	**SHR (95%CI)**	**p-value**	**aSHR (95%CI)**	**p-value**	**aSHR (95%CI)**	**p-value**
Age, year	1.03 (1.02-1.05)	**<0.001**	1.02 (1.00-1.04)	**0.035**	1.02 (1.00-1.04)	**0.013**
HVPG, mmHg	1.11 (1.08-1.15)	**<0.001**	1.07 (1.04-1.11)	**<0.001**	1.06 (1.02-1.10)	**0.004**
CTP score						
A	1		1		-	-
B	4.49 (3.20-6.28)	**<0.001**	2.20 (1.44-3.34)	**<0.001**	-	-
C	6.17 (3.65-10.41)	**<0.001**	2.07 (1.00-4.27)	**0.049**	-	-
UNOS MELD (2016) score, point	1.11 (1.07-1.14)	**<0.001**	-	-	0.98 (0.94-1.03)	0.510
Decompensated vs. compensated ACLD	5.70 (3.97-8.17)	**<0.001**	-	-	2.74 (1.80-4.18)	**<0.001**
Sodium, mmol x L^{-1}	0.90 (0.86-0.93)	**<0.001**	0.97 (0.93-1.02)	0.300	-	-
Creatinine, mg x dL^{-1}	2.71 (1.66-4.41)	**<0.001**	1.56 (0.82-2.99)	0.180	-	-
Albumin, g x L^{-1}	0.89 (0.87-0.91)	**<0.001**	-	-	0.95 (0.91-0.98)	**0.002**
CRP, mg x L^{-1}	2.28 (1.75-2.96)	**<0.001**	1.51 (1.10-2.08)	**0.012**	1.49 (1.07-2.08)	**0.018**
NH3-ULN ≥1.4 vs. <1.4	4.11 (2.74-6.16)	**<0.001**	2.22 (1.27-3.90)	**0.005**	2.09 (1.23-3.55)	**0.006**

Table S4. Competing risk regression analyses of factors associated with hepatic decompensation/liver-related death including – among other parameters – CTP score, serum sodium, and creatinine **(model 1)** or UNOS MELD (2016) score, clinical stage, and serum albumin **(model 2)** with requirement of liver transplantation/non-liver-related death/removal of the primary etiological factor as competing risks in the **outcome cohort**.

Abbreviations: ACLD advanced chronic liver disease; aHR adjusted hazard ratio; NH3-ULN ammonia adjusted for the upper limit of normal; CI confidence interval; CRP C-reactive protein; CTP Child-Turcotte-Pugh score; HVPG hepatic venous pressure gradient; UNOS MELD (2016) United Network for Organ Sharing Model of End-stage Liver Disease (2016)

Table S5

Patient characteristics	**Univariable**		**Model 1 (incl. CTP score, sodium, and creatinine)**		**Model 2 (incl. MELD, and albumin)**	
	**HR (95%CI)**	**p-value**	**aHR (95%CI)**	**p-value**	**aHR (95%CI)**	**p-value**
Age, year	1.04 (1.03-1.06)	**<0.001**	1.04 (1.03-1.06)	**<0.001**	1.04 (1.02-1.05)	**<0.001**
HVPG, mmHg	1.10 (1.07-1.13)	**<0.001**	1.05 (1.02-1.08)	**0.001**	1.03 (1.00-1.07)	**0.046**
CTP score						
A	1		1		-	-
B	3.39 (2.41-4.77)	**<0.001**	2.07 (1.38-3.11)	**<0.001**	-	-
C	5.77 (3.57-9.34)	**<0.001**	3.45 (1.90-6.24)	**<0.001**	-	-
UNOS MELD (2016) score, point	1.11 (1.08-1.14)	**<0.001**	-	-	1.02 (0.98-1.06)	0.392
Decompensated vs. compensated ACLD	4.44 (3.11-6.34)	**<0.001**	-	-	2.37 (1.57-3.57)	**<0.001**
Sodium, mmol x L^{-1}	0.91 (0.88-0.94)	**<0.001**	0.97 (0.93-1.02)	0.226	-	-
Creatinine, mg x dL^{-1}	2.08 (1.22-3.55)	**0.007**	1.23 (0.71-2.13)	0.451	-	-
Albumin, g x L^{-1}	0.90 (0.88-0.92)	**<0.001**	-	-	0.94 (0.91-0.97)	**<0.001**
CRP, mg x L^{-1}	2.00 (1.58-2.53)	**<0.001**	1.20 (0.90-1.61)	0.221	1.16 (0.87-1.55)	0.326
NH3-ULN ≥1.4 vs. <1.4	3.12 (2.03-4.81)	**<0.001**	1.86 (1.17-2.95)	**0.008**	1.78 (1.14-2.79)	**0.011**
**Concordance ± SE**			**0.770 ± 0.020**		**0.788 ± 0.017**	
**AIC**			**1657.052**		**1639.358**	

Table S5. Uni- and multivariable Cox regression analyses of factors associated with non-elective liver-related hospitalisation/liver-related death including – among other parameters – CTP score, serum sodium, and creatinine **(model 1)** or UNOS MELD (2016) score, clinical stage, and serum albumin **(model 2)** in the **outcome cohort**.

Abbreviations: ACLD advanced chronic liver disease; aHR adjusted hazard ratio; AIC Akaike information criterion; AMM-ULN ammonia adjusted for the upper limit of normal; ARLD alcohol-related liver disease; CI confidence interval; CRP C-reactive protein; CTP Child-Turcotte-Pugh score; HVPG hepatic venous pressure gradient; NAFLD non-alcoholic fatty liver disease; SE standard error; UNOS MELD (2016) United Network for Organ Sharing Model of End-stage Liver Disease (2016)

Table S6

Patient characteristics	**Univariable**		**Model 1 (incl. CTP score, sodium, and creatinine)**		**Model 2 (incl. MELD, and albumin)**	
	**SHR (95%CI)**	**p-value**	**aSHR (95%CI)**	**p-value**	**aSHR (95%CI)**	**p-value**
Age, year	1.04 (1.02-1.06)	**<0.001**	1.04 (1.02-1.06)	**<0.001**	1.04 (1.02-1.05)	**<0.001**
HVPG, mmHg	1.07 (1.05-1.10)	**<0.001**	1.03 (0.99-1.06)	0.072	1.01 (0.98-1.05)	0.450
CTP score						
A	1		1		-	-
B	3.31 (2.32-4.73)	**<0.001**	2.08 (1.32-3.30)	**0.002**	-	-
C	5.62 (3.27-9.66)	**<0.001**	3.31 (1.67-6.57)	**<0.001**	-	-
UNOS MELD (2016) score, point	1.09 (1.06-1.13)	**<0.001**	-	-	1.01 (0.96-1.06)	0.840
Decompensated vs. compensated ACLD	4.45 (3.07-6.47)	**<0.001**	-	-	2.61 (1.61-4.22)	**<0.001**
Sodium, mmol x L^{-1}	0.91 (0.87-0.95)	**<0.001**	0.97 (0.92-1.02)	0.230	-	-
Creatinine, mg x dL^{-1}	2.40 (1.40-4.12)	**0.002**	1.35 (0.69-2.66)	0.390	-	-
Albumin, g x L^{-1}	0.91 (0.88-0.93)	**<0.001**	-	-	0.94 (0.91-0.98)	**<0.001**
CRP, mg x L^{-1}	1.92 (1.47-2.51)	**<0.001**	1.24 (0.91-1.69)	0.180	1.21 (0.87-1.68)	0.250
NH3-ULN ≥1.4 vs. <1.4	3.81 (2.46-5.90)	**<0.001**	2.06 (1.18-3.61)	**0.011**	2.12 (1.28-3.50)	**0.004**

Table S6. Competing risk regression analyses of factors associated with non-elective liver-related hospitalisation/liver-related death including – among other parameters – CTP-score, serum sodium, and creatinine **(model 1)** or UNOS MELD (2016) score, clinical stage, and serum albumin **(model 2)** with requirement of liver transplantation/non-liver-related death/removal of the primary etiological factor as competing risks in the **outcome cohort**.

Abbreviations: ACLD advanced chronic liver disease; aHR adjusted hazard ratio; NH3-ULN ammonia adjusted for the upper limit of normal; CI confidence interval; CRP C-reactive protein; CTP Child-Turcotte-Pugh score; HVPG hepatic venous pressure gradient; UNOS MELD (2016) United Network for Organ Sharing Model of End-stage Liver Disease (2016)

Table S7

Patient characteristics	**Univariable**		**Model 1 (incl. CTP score, sodium, and creatinine)**		**Model 2 (incl. MELD, and albumin)**	
	**HR (95%CI)**	**p-value**	**aHR (95%CI)**	**p-value**	**aHR (95%CI)**	**p-value**
Age, year	1.03 (1.01-1.05)	**0.012**	1.02 (1.00-1.04)	**0.036**	1.03 (1.01-1.05)	**0.007**
HVPG, mmHg	1.03 (0.99-1.07)	0.125	1.00 (0.96-1.04)	0.944	1.00 (0.96-1.04)	0.887
CTP score						
A	1		1		-	-
B	1.76 (1.07-2.89)	**0.026**	1.52 (0.89-2.60)	0.122	-	-
C	3.22 (1.79-5.79)	**<0.001**	2.93 (1.46-5.87)	**0.002**	-	-
UNOS MELD (2016) score, point	1.08 (1.04-1.12)	**<0.001**	-	-	1.07 (1.02-1.11)	**0.003**
Sodium, mmol x L^{-1}	0.96 (0.91-0.99)	**0.048**	0.99 (0.94-1.04)	0.549	-	-
Creatinine, mg x dL^{-1}	2.36 (1.34-4.14)	**0.003**	1.93 (1.02-3.65)	**0.045**	-	-
Albumin, g x L^{-1}	0.95 (0.92-0.97)	**<0.001**	-	-	0.97 (0.94-1.01)	0.147
CRP, mg x L^{-1}	1.36 (1.02-1.82)	**0.039**	1.08 (0.77-1.50)	0.668	1.13 (0.81-1.59)	0.473
NH3-ULN ≥1.4 vs. <1.4	1.91 (1.18-3.09)	**<0.001**	1.60 (0.96-2.65)	0.071	1.71 (1.05-2.80)	**0.031**
**Concordance ± SE**			**0.683 ± 0.029**		**0.689 ± 0.029**	
**AIC**			**954.323**		**949.870**	

Table S7. Uni- and multivariable Cox regression analyses of factors associated with the development of acute-on-chronic liver failure (ACLF)/requirement of liver transplantation/liver-related death including – among other parameters – CTP score, serum sodium, and creatinine **(model 1)** or UNOS MELD (2016) score and serum albumin **(model 2)** in patients who already have experienced decompensation at baseline in the **outcome cohort**.

Abbreviations: ACLD advanced chronic liver disease; aHR adjusted hazard ratio; AIC Akaike information criterion; NH3-ULN ammonia adjusted for the upper limit of normal; ARLD alcohol-related liver disease; CI confidence interval; CRP C-reactive protein; CTP Child-Turcotte-Pugh score; HVPG hepatic venous pressure gradient; NAFLD non-alcoholic fatty liver disease; SE standard error; UNOS MELD (2016) United Network for Organ Sharing Model of End-stage Liver Disease (2016)

Table S8

Patient characteristics	**Univariable**		**Model 1 (incl. CTP score, sodium, and creatinine)**		**Model 2 (incl. MELD, and albumin)**	
	**SHR (95%CI)**	**p-value**	**aSHR (95%CI)**	**p-value**	**aSHR (95%CI)**	**p-value**
Age, year	1.02 (1.00-1.04)	**0.022**	1.02 (1.00-1.04)	0.051	1.03 (1.01-1.05)	**0.015**
HVPG, mmHg	1.02 (0.98-1.05)	0.360	0.99 (0.95-1.03)	0.560	0.98 (0.94-1.02)	0.410
CTP score						
A	1		1		-	-
B	1.65 (0.99-2.74)	0.053	1.42 (0.82-2.46)	0.220	-	-
C	3.28 (1.82-5.91)	**<0.001**	2.92 (1.42-6.02)	**0.004**	-	-
UNOS MELD (2016) score, point	1.09 (1.05-1.13)	**<0.001**	-	-	1.08 (1.03-1.13)	**0.003**
Sodium, mmol x L^{-1}	0.94 (0.89-0.99)	**0.032**	0.97 (0.91-1.03)	0.270	-	-
Creatinine, mg x dL^{-1}	2.36 (1.24-4.50)	**0.009**	2.03 (0.93-4.46)	0.077	-	-
Albumin, g x L^{-1}	0.95 (0.92-0.98)	**<0.001**	-	-	0.98 (0.94-1.02)	0.240
CRP, mg x L^{-1}	1.38 (1.02-1.87)	**0.035**	1.11 (0.78-1.57)	0.560	1.21 (0.86-1.71)	0.270
NH3-ULN ≥1.4 vs. <1.4	2.16 (1.36-3.45)	**0.001**	1.80 (1.06-3.06)	**0.030**	1.93 (1.16-3.21)	**0.011**

Table S8. Competing risk regression analyses of factors associated with the development of ACLF/requirement of liver transplantation/liver-related death including – among other parameters – CTP-score, serum sodium, and creatinine **(<u>model 1</u>)** or serum albumin, and UNOS MELD (2016)-score **(<u>model 2</u>)** with non-liver-related death/removal of the primary etiological factor as competing risks in patients with who have experienced decompensation prior to study inclusion in the **<u>outcome cohort</u>**.

Abbreviations: ACLD advanced chronic liver disease; ACLF acute-on-chronic liver failure; aHR adjusted hazard ratio; NH3-ULN ammonia adjusted for the upper limit of normal; CI confidence interval; CRP C-reactive protein; CTP Child-Turcotte-Pugh score; HVPG hepatic venous pressure gradient; UNOS MELD (2016) United Network for Organ Sharing Model of End-stage Liver Disease (2016)
